# OracleTrust: A dual-layer provenance-based signature verification scheme for preventing transaction malleability in blockchain

**DOI:** 10.1371/journal.pone.0348864

**Published:** 2026-05-22

**Authors:** Muhammad Rashid Majeed, Peiyun Zhang, Zeeshan Raza, Thoraya N. Alharthi, Ilyas Khan

**Affiliations:** 1 School of Computer Science, Nanjing University of Information Science and Technology, Nanjing, China; 2 Department of Computer Science, COMSATS University Islamabad, Sahiwal Campus, Pakistan; 3 Department of Mathematics, College of Science, University of Bisha, Bisha, Saudi Arabia; 4 Department of Mathematics, College of Science Al-Zulfi, Majmaah University, Al-Majmaah, Saudi Arabia; 5 Department of Mathematical Sciences, Saveetha School of Engineering, SIMATS, Chennai, Tamil Nadu, India; 6 Hourani Center for Applied Scientific Research, Al-Ahliyya Amman University, Amman, Jordan; 7 Széchenyi István University, Győr, Hungary; Northwestern Polytechnical University School of Software and Microelectronics, CHINA

## Abstract

Decentralized oracle networks pose significant security risks to blockchain systems due to transaction malleability, which can lead to double-spending and integrity issues. While existing solutions such as DAON, SegWit, and SecPLF improve specific aspects of security, they do not address Oracle-driven transaction malleability on a transaction level. DAON focuses on decentralized oracle consensus and reputation mechanisms, but it does not support the cryptographic binding of Oracle metadata to transactions. SegWit reduces signature malleability at the Bitcoin protocol level, but it does not protect the integrity of Oracle-fed data or require validation before transactions are added to the blockchain. SecPLF protects loanable-fund protocols from Oracle manipulation, but it lacks a comprehensive transaction-level solution to prevent Oracle-driven malleability. OracleTrust, on the other hand, uses a dual-layer scheme to bind Oracle metadata and signatures to transactions via provenance tracking and a smart contract validation layer. The first layer encodes transactions into verifiable provenance records, and the second layer dynamically verifies these records with salted Keccak hashing and ECDSA recovery to bind the Oracle signature. A time-constrained commit-reveal mechanism with penalty enforcement ensures that the data is tamper-resistant. OracleTrust outperforms existing solutions in detecting malleable transactions, reducing latency, and memory consumption. This demonstrates its superior robustness and efficiency in blockchain.

## Introduction

Blockchain-based technology is increasingly adopted across a wide range of real-world applications, particularly for financial transactions. However, blockchain transactions can be modified in different ways, introducing malleability and allowing altered transactions to be in the network. If an attacker floods the blockchain network with such transactions, the core of the blockchain becomes overloaded while processing them, leading users to believe their transactions are not confirmed. For example, a genuine owner is fraudulently deprived of assets when a malicious user intentionally discontinues further propagation of the block containing the transaction that moves the token from the buyer’s address to the seller’s address. This work proposes an architecture-level solution to these malleability transaction attacks originating from decentralized oracles, as such situations are increasingly common in blockchain networks [1,2]. To address these issues, this paper first examines the fundamental cause of transaction malleability, especially as it pertains to decentralized oracles.

### Security schemes for untrusted data

To address these challenges, several security schemes for secure blockchain from incoming data have been proposed in recent years, which can be primarily categorized into four types: secure channel construction, decentralized autonomous oracle networks (DAON) [3], truth discovery mechanisms [4], and secure protocols for loanable funds SecPLF [5]. Establishing a secure channel ensures a secure transfer of information through creating strong communication channels between a blockchain system and other data sources in order to guarantee information integrity and prevent any information alterations while it moves across the channels. Nonetheless, securing the channels is not sufficient, as there is still trust issue with data. To address the problem, DAON uses distributed solutions that rely on consensus algorithms and reputation systems, providing Byzantine fault tolerance in oracle networks through non-interactive procedures. Apart from this, truth discovery mechanisms increase the reliability of oracles by aggregating oracle outputs and determining the most truthful values. Lastly, secure protocols for loanable funds (SecPLF) follow similar principles and, in particular, focus on efficient verification procedures, which are important for data integrity and reliability. All the approaches described above help to tackle existing problems.

### Blockchain applications and malleability risks

The importance of blockchain technology and decentralized applications is that value can be transferred without needing an intermediary organization to establish trust. This means increased efficiency and convenience while at the same time providing room for innovation in the development of applications [5–7]. Some applications can be decentralized markets [5], decentralized voting systems [6], and decentralized lending [7]. While the blockchain technology has changed the operations of the DApp, the increasing trend of decentralized oracle being used as a source of external data has created a problem of security, particularly in malleability attacks on the transaction. While various approaches have been suggested to solve the issues related to Oracle Trust or blockchain security, they usually focus on providing either a secure channel, decentralized validation, or ensuring data integrity, and do not explicitly include an approach for transaction malleability prevention. One such case is DAON [3], where Oracle trust has been improved via the implementation of decentralized consensus and reputation approach. It is worth nothing that there is no cryptographic binding between metadata, which is produced by Oracle and the related blockchain transaction. The signature malleability problem can be mitigated by implementing SegWit, but the problem regarding the integrity of the information provided by Oracle before recording it on blockchain remains unresolved. SecPLF [5] protects particular DeFi protocols from potential oracle manipulation, but it still fails to offer a comprehensive solution for preventing oracle-based transaction malleability for all blockchain transactions. Furthermore, academic literature that addresses transaction malleability prevention by using cryptographic provenance tracking and bound signature verification approach is quite sparse.

### Problem statement and motivation

To efficiently mitigate transaction malleability and ensure the integrity of oracle source data in blockchain, this work is driven by two main motivations:

1) To prevent transaction malleability through smart-contract-enforced signature binding and cryptographic provenance tracking. Transaction malleability is a serious threat to blockchain reliability, leading to double-spending, smart contract failures, and transactional inconsistencies [8–10].

Several solutions have been proposed to address this issue. For example, DAON [4] employs consensus protocols and reputation mechanisms to enhance oracle trust, but it does not directly resolve metadata or signature mutability. SegWit [11] restructures the transaction format by separating signatures from the transaction body, thereby reducing signature malleability; however, it lacks mechanisms to secure oracle-fed data before submission. SecPLF [5] introduces secure protocols for loanable funds that ensure verifiable transactions; however, it does not defend against pre-submission tampering of oracle metadata. RollStore [12] provides off-chain storage and verification for external data, but its scope remains limited when both transaction metadata and digital signatures are subject to alteration. To address these limitations, this work presents provenance tracking through smart contracts that cryptographically bind oracle-fed data and its associated signatures to blockchain transactions, ensuring that any alteration to the metadata or signatures invalidates the transaction at its origin.

2) To enable provenance tracking and signature verification, it is necessary to ensure that only integrity-preserving transactions are admitted to the ledger. Existing solutions that delay verification can still allow tampered transactions to enter the blockchain [13,14]. The proposed scheme incorporated an adaptive validation block within a smart contract, verifying provenance and digital signatures at the oracle’s data ingestion stage before submitting to the blockchain. This approach excludes invalid transactions and prevents the blockchain ledger from malleable and fraudulent transactions. The proposed scheme employs provenance tracing and the smart contract technology to prevent attacks based on the concept of malleability.

**Fig 1:**
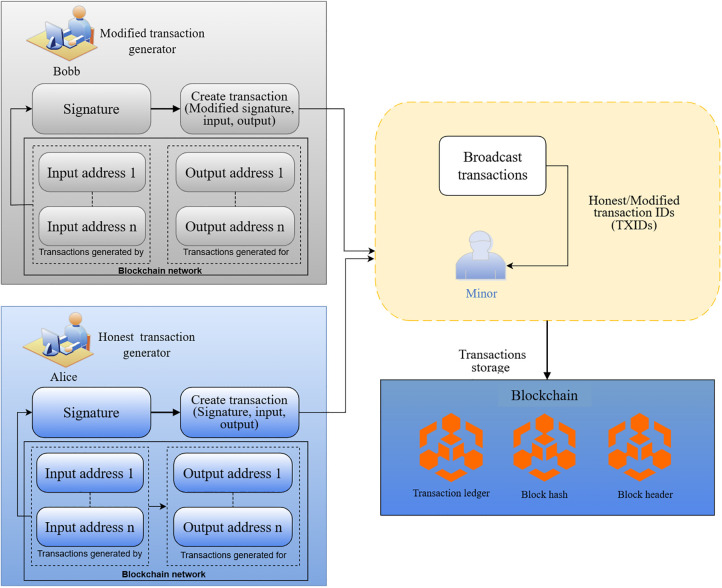
Transaction malleability.

**Fig 2:**
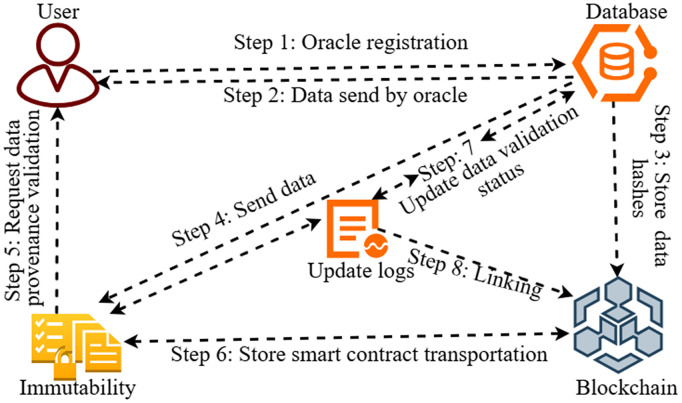
Provenance-based process of data validation.

Fig 1 shows the attack flow, wherein the transaction signature changes while being propagated. As a result, the transaction ID gets changed without changing the transaction nature. The attack starts with (1) the creation of a transaction. In this scenario, Alice creates a transaction with a signature indicating the input and output addresses. Then, the transaction is (2) broadcast to a miner node. During transaction validation, the miner (3) alters the signature (the ECDSA value), but not the actual information. After that, the altered transaction is (4) broadcast back to the mempool and gets (5) a new transaction ID (TXID) due to the signature change. Eventually, the transaction is recorded into the ledger with the new TXID, different from the original one. Thus, an alteration of the signature propagates through the network and causes issues related to malleability, such as, double spending. In contrast, Fig 2 describes a data-validation mechanism using a combination of cryptographic hash functions and smart contracts to validate data. This step goes beyond recognizing malleability by suggesting a prevention method.

### Contributions and organization

Based on the motivations mentioned above, the key contributions of this study are summarized as follows:

- The paper outlines a novel provenance-tracking and a smart contract-based validation model to mitigate transaction malleability and to improve the reliability of oracle-provided data on the blockchain. The proposed provenance tracking, along with smart contract-based validation, aims to verify the validity, authenticity, and integrity of information before it is stored on the blockchain network. The proposed model comprises three vital components of smart contract-based validation, provenance tracking, and cryptographic commitment blocks. The first module records and authenticates the source of each transaction and oracle input; the second module verifies transactions via smart contracts; and the third module comprises a hash-based commitment along with a digital signature to ensure its immutability.
- A bound transaction-validation algorithm is proposed that binds each transaction to a trusted oracle identity using a cryptographic technique. This algorithm leverages Ethereum-compatible digital signature schemes, secure time stamping, and salted hash commitments to ensure the absence of transaction malleability. Each transaction is timestamped at the commitment to ensure traceability and temporal integrity. The signed data is validated using Keccak signature recovery with a set of trusted oracles; if the data is valid, the transactions are executed; otherwise, they are discarded. This algorithm improves transaction integrity, ensures event ordering, reduces the vulnerability of oracle-fed systems, and increases the security of the blockchain network.
- The paper also includes a comparative analysis of OracleTrust in decentralized oracle networks, along with a scalability analysis. Such as DAON, which deals with oracle consensus and reputation; SegWit, which separates signatures in the Bitcoin protocol; and SecPLF, which provides security to DeFi protocols, OracleTrust is the only system that deals with preventing oracle-driven transaction malleability at the transaction level through a dual-layer architecture, along with provenance formation and smart contract validation. The dual-layer architecture of OracleTrust has been tested under high transaction volumes and across different oracle participation rates to show its scalability in decentralized oracle networks without compromising security.

The rest of the paper is organized as follows. The Related Work section reviews research relevant to this study. The Preliminaries section introduces the necessary background concepts. The Proposed Model section outlines the proposed model, architecture, framework, threat analysis, design goals, and security assumptions. The Proposed Scheme section presents a detailed description of the proposed scheme. The Algorithm and Security Analysis section presents the proposed algorithms and discusses the security analysis. The Experimental section describes the experimental setup and provide a detailed discussion of the results. Finally, the Conclusion section summarizes the findings and outlines future research directions.

### Related work

On-chain provenance is critical to preserving the integrity of blockchain transactions. It provides an immutable history of any data exchange. Recent work has focused on cryptographic methods such as hashing and digital signatures to verify the authenticity of recorded data. For example, a new method is presented in [13] that uses secure hashing algorithms to verify the integrity of transactions on data in decentralized networks. In the same manner, [15] developed an improved version of a blockchain-based multi-signature scheme that extends time-stamping and multi-signature techniques, ensuring that the blockchain remains immutable while maintaining the relationship between data exchanges. However, even with the recent developments, scalability remains a problem, particularly in low-volume applications such as the Internet of Things (IoT) networks [16,17]. The development of blockchain oracles is increasingly influenced by the architectural and philosophical differences between major blockchain ecosystems. Although Ethereum defined the initial model of smart contract-interconnected oracles, new networks, such as Polkadot, Cardano, and Solana, have developed their own models of scalability, consensus, and interoperability, each with its own oracle systems. These architectural differences affect the design trade-offs and the security vulnerability of the oracle networks, especially on transaction malleability, data integrity, and cross-chain data validation.

### Polkadot ecosystem

The Polkadot platform is based on a heterogeneous multichain architecture, where parachains are linked to one common relay chain, thus enabling cross-chain oracle messaging and security. As per literature, decentralized oracle protocols can be treated as cross-chain bridges that facilitate the interoperability of data transfer between different blockchains autonomously, which is crucial while using DeFi in practical applications (for instance, cross-blockchain message delivery mechanisms introduced in [18]). Nevertheless, data consistency and trust issues between independent parachains remain significant priorities.

### Solana oracle environment

Solana prioritizes high throughput and finality under one second, enabling low-latency oracle integrations through protocols such as Pyth Network. However, many oracle deployments on Solana rely on committee validation or privileged nodes, which centralize trust and increase the risk of collusion and data manipulation, as reported in recent infrastructure studies [19,20].

### Ethereum oracle ecosystem

Ethereum continues to maintain the most mature oracle ecosystem. For instance, the Chainlink cross-chain interoperability protocol (CCIP) was released in 2023 and continues to expand secure cross-chain messaging and data feeds across more than 50 blockchains [21]. The recent empirical literature points to deviations in Chainlink price feeds under market stress (depending on heartbeat- and threshold-related oracle design) and the significance of oracle design decisions in DeFi for reliability and economic effects. In fact, recent cross-platform [22] research demonstrates that, in the case of smart contract functionality, it is possible to deploy and execute the same smart contract on different public blockchain systems, such as Ethereum, Tezos, Polkadot, and Solana, by re-implementing the smart contract logic in the native programming language of the specific blockchain systems under consideration. The philosophy behind the design and implementation of OracleTrust, it is submitted, is similar; on the one hand, the overall provenance and signature validation logic is defined at the smart contract level, and, on the other, the overall infrastructure, such as a cross-blockchain adapter, is responsible for mapping this overall logic onto the specific execution environments defined by Ethereum, Polkadot, and Solana. In such ecosystems, a common issue is that, through existing oracle solutions, data correctness and transaction integrity are treated as distinct issues. The majority of protocols focus on securing oracle data delivery and cryptographic attestation, but not on cryptographically binding the data to the transaction that uses it and spending such data at the consensus layer with enforceable timeliness and non-repudiation guarantees. Consequently, adversaries can exploit temporal discrepancies, replay attacks, or signature malleability even when the source data is valid, undermining the finality and reliability of smart contract execution [23,24].

### Blockchain security, interoperability, and trust

The capacity to build trust in the integrity of data and transactions through a consensus mechanism is one of the essential properties of blockchain, which requires that transactions be validated and approved by a majority of the network participants. Still, reaching the truth is a more complicated issue, and that is where the idea of oracles comes to play [17]. Recent developments in blockchain technology have focused on interoperability between different blockchains, which is highly critical in enhancing the scalability and functionality of the decentralized networks. The current developments in blockchain security and interoperability have been geared toward addressing weaknesses across different levels of blockchain architecture and facilitating the smooth interaction of different blockchain networks. According to [25], cryptographic schemes and strategies, such as asymmetric encryption and timestamping, are crucial for safeguarding against alteration and improving trust in blockchain applications. They also address critical attack vectors, such as 51% attacks, and propose solutions, such as trust-based consensus, to enhance against attacks. In addition to the delivery of the blockchain, [26] proposed a cross-chain architecture for digital resources, founded on the relay chain technology, to facilitate the safe purchase and sale of assets between two blockchain systems. It is scalable, supports additional functionality, such as DDoS attacks and single-point failures, and provides an assurance of message relay between parallel chains, as it is secure. The combined focus on security and interoperability improves the integrity and scalability of blockchain systems. Another cross-chain oracle solution, such as those based on LayerZero or Wormhole [27,28], focuses on interoperability and data relay across chains. While crucial for multi-chain applications, their designs optimize for message-passing efficiency and often rely on external validator committees. This introduces additional latency and trust assumptions without providing a mechanism for transaction-level binding or temporal enforcement on the destination chain, making them unsuitable for preventing oracle-driven malleability. However, it still faces security and trust challenges across multiple levels, including consensus, network, smart contracts, and applications. Such attacks on consensus mechanisms and data propagation highlight open areas that require further study to enhance blockchain security [29].

### Malleability attack protection approaches and protocol-based mitigation

Most current methods in enhancing the security of blockchain technology usually do not consider transaction malleability in particular when it comes to blockchains that employ decentralized oracles. Despite the high levels of cryptographic security offered by core blockchain protocols (such as Bitcoin), the applications built on top of them present exploitable gaps in security that can be breached via malleable transactions and thus cause confirmation delays and data tampering [30]. Prior literature [29,31], and [32,33] has indicated the changes to the rules concerning transaction spending in order to eliminate the problem of transaction malleability in decentralized computing protocols, though not because it means that the oracle layer is not prone to vulnerabilities. What these changes do not tackle is any vulnerability stemming from the oracle layer. For example, Chainlink before SegWit implementation [20] had the problem of signature-based validation, though it was still vulnerable to transaction ID manipulation, impacting the integrity of the oracle update process. Another example of an oracle-based application susceptible to malleability attacks is Town Crier [34,35], which employs the TEE framework for external input provision, but due to use of the standard transactions model, is prone to replay and malleability attacks. In the same vein, the new generation of oracle solutions relying on TEE (such as DECO [36]) improves on the data attestation problem but leaves data transmission vulnerable.

In the context of protocol-based approaches, NewSCS [32] improves a fair two-party protocol by using each party’s secret and the other party’s signature to prevent the malleability attacks on the Fuse transaction. Specifically, one A’s transaction *Fuse*^*A*^ (in: $Commit^{A}$) redeems its transaction $Commit^{A}$ (in: *T*^*A*^) by providing the signature script with the signature *sig*_*B*_ [37] and the revealed secret *Sr*_*A*_. Another party B’s transaction *Fuse*_*B*_ (In: $Commit_{B}$) redeems its transaction Commit B (in: *T*^*B*^) by providing the signature script with the signature *sig*_*A*_ [26] and the revealed secret *Sr*_*B*_. Every party is required to perform another round of the commit protocol to disclose their secret *r*_*A*_ or *r*_*B*_, which introduces high complexity to NewSCS. Maximum attention to potential vulnerabilities is required to guarantee the validity of protocols. Likewise, a timed-commitment approach is proposed in [38] to mitigate transaction malleability in storage through a deposit protocol. When the depositing method commits an unmodifiable transaction, the compensation system allows the protocol to proceed with all activities based on activated transactions. The idea behind this solution is to encourage additional protocols to protect malleability. A ‘*Fuse*’ transaction attack in the commitment scheme exposes the committer to the risk of at least double penalties by sequentially triggering two separate ‘*Fuse*’ attack, thereby introducing an additional vulnerability.

### Cryptographic defenses and future challenges

Transaction malleability is a major threat to blockchain security, promoting researchers to suggest a range of cryptographic defenses [25,39]. One such method is the threshold signature scheme (ECDSA) [38,40], which distributes signing authority among multiple parties to prevent key theft and unauthorized modification of transactions. This scheme is widely adopted in cryptocurrencies like Cardano and Decred to enhance transaction security [25]. Moreover, these attacks can be perpetrated using signatures, which need further safeguarding. To resolve this issue, post-quantum proof-of-work PoW algorithms have been proposed, where the hardness of the signature is adjusted according to the probability of producing a legitimate signature. Some of the latest approaches to crypto solutions include zkOracle [41], where a zero-knowledge proof is used to authenticate computations off-chain, and DORA proposes using dynamic reputation graphs for oracle trust. These are great steps forward in verifiability and long-term accountability. Nevertheless, they frequently treat data correctness and transactions security as two distinct issues; as studied in recent surveys [42,43], they often treat data correctness and transaction security as separate concerns. A well-reputed oracle in a DORA-like system could still manipulate transaction outcomes by controlling the timing of its data reveal, as its reputation score may not incorporate provenance tracking enforced at the smart contract level. Consequently, there is a significant gap of existing solutions in the form of protocol level malleability patches (NIPut, IIS), decentralized oracle networks (Chainlink, Band), TEE-based attestation (Town Crier, DECO), or new trust models (zkOracle, DORA) do not have a framework that cryptographically binds oracle data to a particular transaction, places hard time requirements on data reveals, or verifies the binding prior to ledger admission. This loophole enables oracle-induced transaction malleability, in which the provenance and timeliness of the data are not part of the transaction validity check. Moreover, the interactive inclusion signature scheme has been introduced to provide non-repudiation and verifiable transaction inclusion in the blockchain to prevent any possible modifications of the transactions by some fraudster [44]. However, despite providing enhanced security, there is a range of factors that may impede a wide adoption of these security schemes including significant computational expenses and network latency. Apart from the mentioned security systems, various other schemes have been proposed aimed at addressing transaction malleability. NIput [45] proposes a rather simple and efficient approach to prevent malleability attacks for Bitcoin through changing the way the transaction ID is computed. Incontestable Signatures (IIS) [46] propose interactive protocols where transaction owner interacts with the producer of the transaction to make it incontestable and provide nearly instant confirmation. However, it can be attacked with malleability-based attacks, as discussed in SigNT [47], therefore, the attacks on the transactions in these protocols need to be considered as well.

## Provenance

The concept of data provenance, commonly known as lineage, involves recording the origin of data and its path during the lifecycle. It includes metadata [48,49] that set out the origins, history, and evolution of an end product. Provenance involves a series of data, processes, activities, and users associated with data, collectively known as the data lifecycle. The provenance is very important in such supply chains, digital forensics, and scientific collaboration [48]. Provenance is crucial in scientific research collaborations, but current systems struggle to ensure data security and collaboration. which emphasizes the growing need for tamper-proof storage of scientific data provenance to facilitate data sharing. Several studies propose methods to manage scientific workflow provenance. For example, Blockflow [50].

Table 1 shows a comparative analysis of existing methods and the proposed method across various factors. The existing methods, such as the accessible signature scheme, digital signature-based authentication, and SegWit, address transaction malleability prevention to some extent, but only the proposed method significantly reduces it. Though certain techniques, such as DAON and SecPLF, provide partial security or none at all of the oracle, the proposed technique effectively mitigates these issues. Whereas some of them have adopted validation in their systems, such as digital signature-based authentication, DAON, SegWit, and SecPLF, RollStore does not. Conversely, OracleTrust combines a validation process that guarantees instant verification of oracle information with blocking tampered transactions before they are written on the blockchain. On scalability, the techniques span a spectrum ranging from low scalability in the Accessional signature scheme, digital signature-based authentication, and DAON to high scalability in RollStore and the proposed method. In terms of security, SegWit and RollStore provide higher security, while others like the Accessional signature scheme, digital signature-based authentication, and DAON offer moderate security. The proposed method ensures high security in all aspects.

**Table 1:** Comparative analysis of existing methods and the proposed method.

Scheme	Transaction malleability prevention	Decentralized oracle security	oracle validation	Scalability	Security	Smart contract-based validation
Accessional signature scheme [51]	Yes	No	No	Low	Moderate	No
Digital signature-based authentication [52]	Yes	No	No	Low	Moderate	No
DAON [3]	No	Yes	Yes	Low	Moderate	Yes
SegWit [53]	Yes	No	No	Moderate	High	No
SecPLF [5]	No	Partial	Yes	Moderate	Moderate	Partial
RollStore [12]	No	No	No	High	Low	No
Proposed method	Fully prevented	Yes	High	High	High	High

### Provenance in supply chain and blockchain solutions

Blockchain studies of supply chains have so far been classified across numerous fields, with the pharmaceutical industry a major player in the global supply chain. One of these regulates the production and manufacture, as well as the marketing, of essential medicines and healthcare products. Stakeholders in the pharmaceutical supply chain include producers, distributors, pharmacies, and healthcare providers [54]. Effective management is crucial for address challenges related to coordination, communication, procurement, storage, shipping, and regulatory compliance. Proper management is important for addressing issues of coordination, communication, procurement, storage, shipping, and regulatory compliance [32]. The concept of provenance holds particular significance in scientific workflows, with various works like BlockFlow, SciLedger, Smart Provenance, DataProv, SciBlock, Bloxberg, and SciChain introducing specialized approaches incorporating event listeners, voting systems, decentralized databases, timestamp-based invalidation, and unique provenance models [55,56]. For example, Nguyen et al. [30,57] combined IoT edge orchestration with blockchain to enhance trust, while Faraj et al. introduced BlockPro [58] for secure IoT data provenance. Akbarfam et al. [48] proposed a blockchain framework for cloud-centric IoT to identify data origins. In digital forensics, provenance records are vital for evidence integrity, but the IoTFC framework faces limitations in access control and component communication. Recent solutions such as Forensiblock [48], a private blockchain with a forensics focus, and a Hyperledger-based trust system [59] for tracking media files as evidence aim to address these challenges.

Existing methods for preventing transaction malleability and oracle validation, such as DAON and SegWit, provide partial support for smart contract-based validation and lack integrated provenance tracking. These methods do not provide systematic protection, especially against manipulated oracle-fed data.

To improve transaction integrity and ensure stable oracle interaction, this work develops an innovative provenance-based technique that combines smart contract-based validation with cryptographic provenance tracking. The proposed has metadata validation, signature verification, and timestamp-based signature verification to check integrity. The proposed method combines these mechanisms to increase resistance to transaction malleability, improve system scalability, and provide high-level validation in decentralized environments.

## Preliminaries

The Poisson distribution can be used to estimate the potential progress or effort an attacker can make. In this scenario, the acknowledgment for data is withheld until the transaction becomes part of a confirmed block, and additional *z* blocks are appended, ensuring that honest blocks are added at the average expected time per block [8,51,57]. Let λ represent the progress of the attacker that is predicted:


$$\begin{array}{c} \lambda =z*\left(\frac{q}{p}\right) \end{array}$$ (1)

where it *p* represents the chance of an honest miner to find the next block and *q* denotes the chance and the mining power of the attacker to find the next block. The transaction’s progress is then computed by calculating the interval *n* at which the adversary can make progress on altering the transaction:


$$\begin{array}{c} n=z*\left(\frac{t}{p}\right) \end{array}$$ (2)

where *z* represents the number of blocks issued by the attacker, and *t* is the time in minutes it takes for the attacker to produce these blocks. Let *f* represent the rate at which the attacker adds blocks. It is defined as:


$$\begin{array}{c} f=\frac{q}{t} \end{array}$$ (3)

where *q* is the number of blocks produced by the attacker, and *t* is the time in minutes. Eq. (3) helps in understanding the time interval within which the attacker can affect the blockchain by altering transactions. Therefore, the average number of successes λ are expected to occur within the interval will be achieved through a multiplication of the equation, which is the equation, Eq. (1) and Eq. (3) as follow: Applying the interval and rate of addition the attacker does. The progress of the attacker can now be obtained as:


$$\begin{array}{c} \lambda =z*\left(\frac{t}{p}\right)*\left(\frac{q}{t}\right)=\frac{z*q}{p} \end{array}$$ (4)

This equation combines the interval and rate to give an overall prediction for the attacker’s progress in altering transactions on the blockchain. It highlights how the attacker’s hash power *q* and the time taken by honest miners *p* influence the attacker’s ability to alter the blockchain state.

Next, a Poisson distribution is used to model the probability of an attacker achieving a given number of successful modifications to the blockchain.

Let *X* represent the attempts to succeed, where *X* = *k* represent the number of success and *k* is a non-negative integer (*k* ≥ 0), and the probability can be determined as:


$$\begin{array}{c} p(X=k;\lambda )=\frac{\lambda^{k}e^{-\lambda }}{k!} \end{array}$$ (5)

In this equation, p(X=k;λ) represents the probability of *k* successes occurring, where λ is the mean number of occurrences (or blocks added) predicted. This formula helps in estimating the likelihood of a specific number of successful attacks or progressions within a given time frame. Equation (4) and (5) can be used together to help the system successfully identify long-term and instant changes in the state of the blockchain, thus making its a useful tool for analyzing potential attack scenarios occurring in blockchain systems, as is the case in the study [8,47,57].

## Multi-Signature Scheme (MSS)

A multi-signature scheme (MSS) is a cryptographic technique used to protect blockchain transactions against malleability attacks. The scheme operates by aggregating partial signatures from multiple signers into a unified, unmalleable signature. The verification process for MSS is outlined in the following steps:

Let Γ denote the signature of individual participants that are summed to from the overall signature of all participants. The object checked during the verification process is this signature:


$$\begin{array}{c} \Gamma =\sum_{i=1}^{N}\sigma_{i} \end{array}$$ (6)

where *i* is the index variable that runs through each individual signers in the multi-signature scheme [15], starting from 1 and going up to *N*, where *N* is the total number of participants involved in generating the signature. *N* represents the total number of signers (participants) in the multi-signature process, indicating the number of individuals or entities contributing their signatures to the aggregated signature Γ. $\sigma_{i}$ is the individual signature generated by signer *i*, where *i* ranges from 1 to *N*. Each signer produces a partial signature using their private key and the transaction data, and these partial signatures are then aggregated to form the final signature Γ.

Let μ be the challenge value, computed based on the transaction message *M*, the aggregated signature Γ, and the public keys of all signers. The challenge is computed using a cryptographic hash function *H* as follows:


$$\begin{array}{c} \mu =H(M,\Gamma ,pk_{1},pk_{2},\ldots ,pk_{N}) \end{array}$$ (7)

where *M* represents the transaction message, Γ is the aggregated signature, and $pk_{1},pk_{2},\ldots ,pk_{N}$ are the public keys of the signers. This challenge value plays a key role in the verification process, ensuring that the aggregated signature corresponds to the correct transaction data and the public keys of all the participants.

Let *PK*_*agg*_ denote the aggregated public key. The aggregated public key *PK*_*agg*_ is computed by summing the public keys of all signers, as follows:


$$\begin{array}{c} PK_{agg}=\sum_{i=1}^{N}pk_{i} \end{array}$$ (8)

where *pk*_*i*_ is the public key of signer *i*, and *PK*_*agg*_ is the total aggregated public key, which represents the collective contribution of all the participants in the multi-signature scheme.

Let *R* represent the *aggregated nonce point*, obtained by summing the nonce points from all participants in the scheme:


$$\begin{array}{c} R=\sum_{i=1}^{N}R_{i} \end{array}$$ (9)

where *R*_*i*_ denotes the nonce point generated by the *i*-th signer, *i* ranges from 1 to *N*, and *N*. This aggregation ensures that each participant’s nonce contribution of every participant is incorporated into the overall signature generation process.

Let Γ denote the *aggregated signature*, which is the final scalar signature obtained by securely combining the partial signatures contributed by all signers. Let *G* denote the *generator point* on the elliptic curve, a fixed and publicly known point that serves as the foundation of elliptic curve cryptography and plays a critical role in the verification process. The verification equation for the aggregated signature is expressed as:


$$\begin{array}{c} \Gamma \cdot G\overset{?}{=}R+\mu \cdot PK_{agg} \end{array}$$ (10)

In this equation, it μ represents the *challenge value*, derived from the transaction message and the associated cryptographic parameters, ensuring that the signature is strongly bound to the transaction and the participating signers. The term *PK*_*agg*_ is the *aggregated public key*, computed as the sum of the individual public keys of all signers. It represents their collective commitment to the transaction and ensures that the verification process reflects the joint authorization rather than any individual.

Together, Equation (9) and Equation (10) establish the foundation of the multi-signature verification process, where the aggregated nonce, the challenge value, and the aggregated public key collectively guarantee the *integrity and authenticity* of the aggregated signature.

### Cryptographic design rationale

The OracleTrust implementation is based on cryptographic primitives that guarantee security properties like confidentiality and efficiency. For the hash function, Keccak-256 is used. This is due to the fact that it is native to Ethereum and can be implemented in EVM-compliant languages. In general, it is seen as a standard for address generation and other smart contracts implementations [60,61]. When compared to alternative cryptographic hash functions, e.g., SHA-256, Keccak-256 performs better in terms of gas consumption. Moreover, it offers strong resistance to collisions and preimage attacks, making it well-suited for binding provenance and integrity verification [62]. In Oracle authentication, OracleTrust employs ECDSA signature recovery rather than signature verification. ECDSA signature recovery enables the direct derivation of the signer’s blockchain address from the signature. This is similar to the Ethereum identity model based on addresses. Moreover, there is no need to store public keys for OracleTrust since signature recovery is used. This reduces computational and storage costs while at the same time providing strong binding for Oracle identities with submitted data. Authentication based on the elliptic curve discrete logarithm is widely used in blockchain systems that use ECDSA and is deemed to be secure under the elliptic curve discrete logarithm assumption [63]. Although other cryptography schemes, e.g., non-EVM-based signatures and non-EVM-based hash functions, are extensively applied in cryptographic systems, they have been considered and avoided in OracleTrust because of their relatively high computing cost, and suboptimal performance in smart contract-based systems. The entries for the selected cryptographic algorithms, i.e., Keccak-256 and ECDSA with signature recovery, offer the best balance of security and efficiency, a realistic level of deployability, and are especially suitable in scalable and decentralized blockchain applications based on RSA.

## Proposed model

This section describes the proposed model, framework, threat analysis, and design goals, respectively.

### Proposed oracle trust operational model

Fig 2 represents a provenance-based data validation procedure to ensure data authenticity, integrity, and trust within a decentralized system. The process begins with Step 1, where the User initiates the request for data provenance validation (Step 5). This step ensures that the data received from the oracle is genuine and traceable to its origin. This ensures that the information remains authentic and reliable throughout the process. Subsequently, in Step 2, the oracle sends the information to the system. At Step 4, the *T* Blockchain validates the data using digital signatures and hashing techniques to ensure that only untampered information is allowed. After validation of the information, in Step 6, the data moves to the *S* blockchain where smart contracts are used for performing transactions based on the rules established by businesses. The S blockchain ensures data immutability since data cannot be changed after validation. The provenance layer (Step 5) ensures that the integrity and origin of the data are verified before it moves to the next layers. This is complemented by *T* Blockchain for initial data validation and *S* Blockchain for secure execution and transactions. Step 8 involved linking the data provenance and ensuring complete traceability.

Meanwhile, a database archive of Step 3 data hashes is created, enabling integrity to be easily checked. Such a multi-layered solution increases the security, transparency, and reliability of the system, mitigates risk related to malicious oracles and transaction malleability, and guarantees the processing of only authentic data.

### Framework for the provenance- and smart contract-based validation scheme

**Fig 3:**
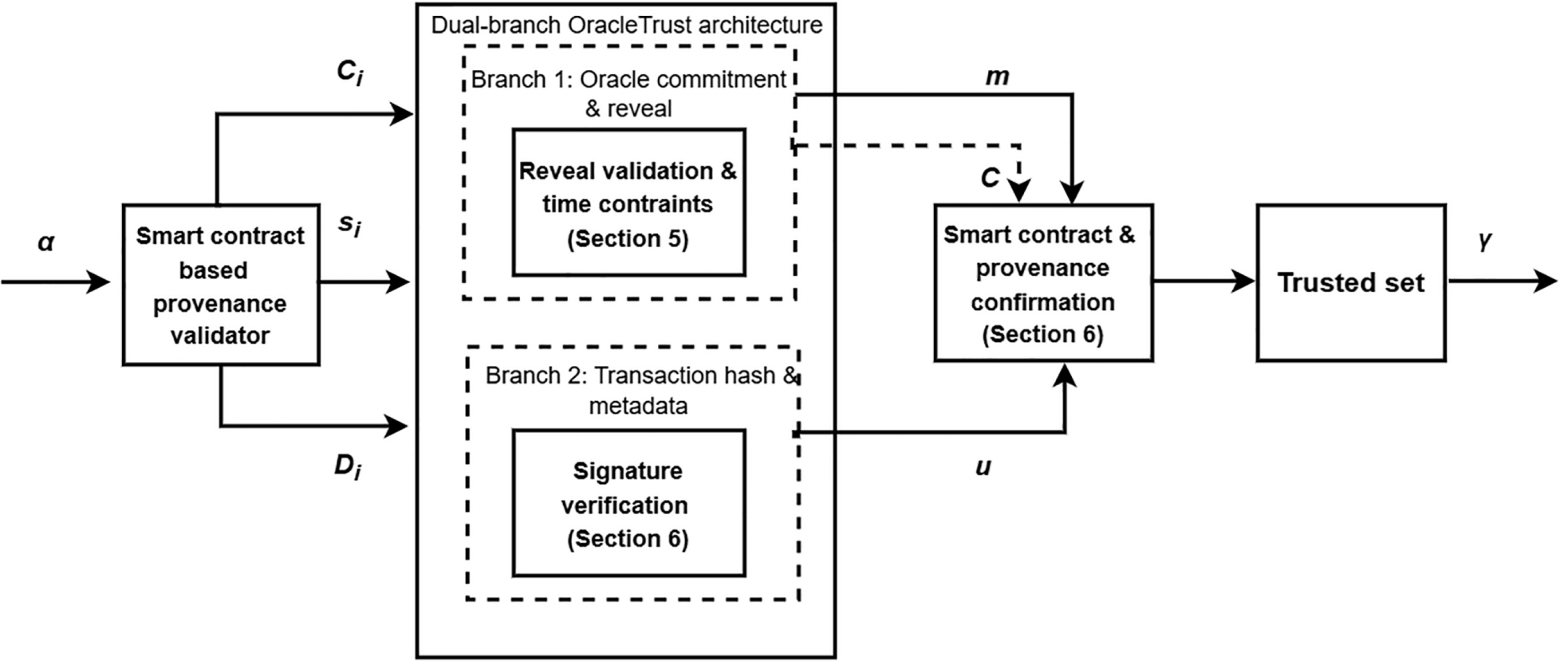
The framework of the proposed model.

Fig 3 provides a detailed view of the proposed validation framework that will be based on the ideas presented in the previous section. It demonstrates how the system leverages provenance-based checks and smart contract validation to achieve the integrity and authenticity of the blockchain transactions. The figure shows how the two parallel validation branches, one that authenticates the oracle data and the other that authenticates the blockchain transaction, interrelate to give the final trust decision. In Fig 3, the proposed model starts with a single input vector, denoted alpha(α), which consists of five elements: the raw blockchain transaction (*T*), the oracle-supplied data (*D*_*i*_), the associated salt (*s*_*i*_), the original commitment hash (*C*_*i*_). These inputs are fed into the main blockchain transaction unit, which then separates them into two parallel validation units. Branch 1 authenticates the oracle information by ensuring that the disclosed pair of information (*D*_*i*_, *s*_*i*_) correctly corresponds to the stored commitment hash (*C*_*i*_) and confirms that this reveal occurs within an authorized time window. Successful validation results in branch 1 authenticated metadata and commitment reveal, which is referred to as *m*. Simultaneously, Branch 2 authenticates the blockchain transaction by computing the hashing of *T* and verifying the signature σ to recover the signer’s address *u*, which is then validated against a trusted set of oracle identities. Both these outputs, *m* and *u*, feed into the smart contract and provenance confirmation module, which consolidates the results and recomputes a new commitment hash *C* with the help of a cryptographic function to verify end-to-end data integrity.

Finally, the module will provide a validation outcome of a transaction, denoted by value of γ, which denotes if the transaction and the Oracle data meet all the provenance and authenticity criteria, thus providing trust within the blockchain network. Although Fig 2 provides a high-level view of how provenance and validation are applied to oracle-fed data, Fig 3 delves deeper into the internal working mechanism of the proposed model. It shows a two-branch architecture: one branch verifies oracle commitments using salts and hashes, and the other verifies the transaction’s cryptographic signature. Both validation streams merge at the smart contract level, where the final trust decision is made. Thus, Fig 3 builds on the reasoning in Fig 2 by describing the dual validation mechanism and the interaction between oracle proofs and transaction signatures.

### Scalability of oracletrust

Scalability becomes a problem in blockchain-based solutions with decentralized oracle networks, which are dependent on the rise of transaction volumes and the amount of data provided to them by oracles. OracleTrust proposes a two-tiered architecture where transaction validation involves off-chain and on-chain processes. During the transaction validation process, the off-chain tier deals with most of the computations and memory consumption from the blockchain side, whereas the on-chain tier obtained through a smart contract ensures the consistency and integrity of validated data. The proposed system offers scalability opportunities through parallelization and aggregation. It reduces computational cost by shifting certain tasks to the off-chain tier, which includes data and signatures validation. In contrast to traditional systems, where the computational overhead of transactions depends on the number of transactions, in OracleTrust, the on-chain load is proportional to the size of succinct proofs (e.g., cryptographic hashes and signatures’ aggregation). For instance, at any given time, the size of these proofs is proportionate to the number of oracle sets (batches) or oracle sets, but not the number of transactions processed. OracleTrust is a scalable solution for decentralised oracle network validation due to transaction verification performed off-chain and on-chain tiers. Besides, besides scalability, the system tackles the practical limitations of network congestion, coordination delay between oracles, and transaction backlogging. In the proposed design, the off-chain tier processes computationally expensive preprocessing tasks, while the on-chain one implements integrity and consistency checks by using a smart contract. In addition, OracleTrust includes mechanisms to address the coordination delay issue. By only accepting verified oracle data, the system eliminates queues and prevents delays in the transaction processing process.

### Architecture for truthful verification and oracle trust

**Fig 4:**
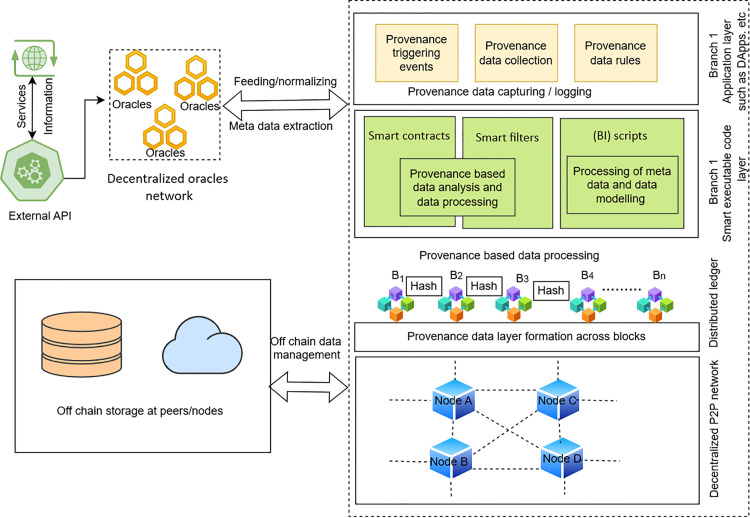
Dual-branch architecture of decentralized oracle-based provenance data processing.

The proposed model architecture is shown in Fig 4. It comprises multiple layers that collaborate seamlessly to address transaction malleability, data provenance, and oracle trust issues. The system uses a multi-layered architecture, comprising external services, a decentralized oracle network, blockchain technology, and off-chain data management.

Below is a detailed breakdown of each component:

**1) External API and services:** The system begins with the extraction of data from the external API, which provides data from different sources. This data is the input of the architecture and is required to initiate the further stages of data processing.
**2) Decentralized oracle network:** At the core of this architecture is the decentralized oracle network. Multiple oracles that feed and normalise data from external API sources. These oracles ensure that incoming data is standardised and prepared for processing within the system. This topology does not allow for individual points of failure that make the system reliable and resilient.
**3) Provenance data capturing and logging:** After receiving the data provided by the oracles, the data is fed into the provenance data capturing layer. This layer documents the triggering events, data collection procedures, and provenance data rules. Data provenance is recorded to maintain a clear, traceable history of data throughout its lifecycle. This plays a vital role in ensuring that the data is authentic and that the source of information is given, since the information as a whole has its own history.
**4) Provenance-based data processing:** The system uses smart contracts and smart filters to process the data according to provenance rules. The smart contracts automatically verify the data, ensuring it complies with the network’s rules and is not tampered with. Metadata is then analyzed by the business intelligence (BI) scripts, and data modeling is done to generate actionable insights. This layer plays a vital role in ensuring that only valid, verified, and reliable data gets processed.
**5) Provenance layer formation and blockchain integration:** When the data is hashed, the cryptographical hashes form the Provenance Data Layer that associates the data with its provenance. The layer plays a vital role in creating a safe, indestructible record of the data that is replicated over distributed ledger systems. Each data block (B1, B2, B3, etc.) is securely linked using these hashes, creating a chain of provenance that is impossible to alter without detection.
**6) Distributed ledger and peer-to-peer (P2P) network**: The data is eventually integrated into the distributed ledger network, which enables the overall architecture to be decentralized. The P2P network enables nodes (Node A-D) to interact with each other and allows the transfer of data confidentially and effectively. This network is decentralized, which increases the system security; it is not centrally controlled or manipulated.
**7) Off-chain data management:** To handle the large amount of data that is produced, the architecture uses off-chain data management. This implies that it can store data at peers/nodes, while the important provenance information and transaction details can be safely stored on the blockchain. Such a hybrid system is the best solution because it maximizes the performance and scalability of the blockchain while ensuring data integrity and availability.

The proposed architecture brings together several innovative features. The system leverages a decentralized oracle network, eliminating the risks posed by malicious data sources and enhancing data trustworthiness.

To secure from malleability attacks on transactions driven by oracle, the architecture incorporates external data sources, a decentralized oracle network, provenance capture, the execution of smart contracts, and on/off-chain storage. External APIs supply raw data to a decentralized oracle network, which normalizes it and forwards it to the provenance data capturing and logging layer. Provenance-tagged data is then processed by smart contracts and smart filters and enriched with business-intelligence scripts that apply application-specific rules. A provenance layer calculates the hashes of the validated records into immutable blocks $B_{1},B_{2},\ldots$, which are stored on a distributed ledger operated by a P2P network of nodes. Off-chain data management at peer nodes handles large datasets, while critical provenance hashes and metadata remain on-chain. The figure illustrates how these components work together in a hybrid on-chain and off-chain design to provide scalable, auditable, and tamper-resistant oracle data validation within OracleTrust. OracleTrust has a two-layer design that isolates transaction validation on off-chain and on-chain layers so that oracle networks (DONs) may be decentralized and scalable. The off-chain validation layer handles most transaction validation, and only verified tamper-proof data is sent to the blockchain. This reduces congestion on the blockchain network, enabling it to effectively handle a higher volume of transactions. The smart contract validation layer on the chain helps ensure data integrity, and only the valid transactions are logged onto the blockchain. The design supports deployment in decentralized oracle networks and other high-volume decentralized applications. Furthermore, a provenance layer will be added to ensure the addition of trustworthy audit trails that increase the level of confidence in the entire system. With the inclusion of blockchain technology, a smart contract will be used to validate and process the data based on pre-established conditions, making the information tamper-proof and traceable since it is executed securely on the blockchain. Additionally, the hashing algorithm guarantees that the information stored within the system is not altered without the owner’s consent and maintains its integrity. Lastly, the system is optimized for efficient data storage and ensures the immutability of blockchain for transaction tracking purposes. Fig 3 builds on the framework of Fig 2 by presenting the internal validation logic of the proposed model. In Fig 3, the emphasis is on validation logic, whereas in Fig 4, the complete system architecture is implemented in practice through components, external APIs, decentralized oracle networks, provenance logging such as a peer-to-peer network, distributed ledger, and hybrid on- and off-chain storage. In this way, Fig 4 scales the framework depicted in Fig 3, which makes it applicable to the real world. The following section presents ‌‌the threat analysis and security model.

### Threat analysis and security model

Let **A** be the adversary targeting a specific blockchain transaction, denoted as *T*. The adversary wants to tamper with the transaction and forge an interactive signature, so that it produces a bogus signature with a significantly different value of $\epsilon^{\prime }$ within the specified consensus time frame τ.

Initially, node *A* receives the transaction *T* message, which is transmitted by honest agents and takes advantage of a security flaw in the communication protocol. In order to go further with this, *A* uses multiple spoofed nodes that masquerade as legitimate network nodes and place them near strategic target nodes. These manipulated nodes then relay a modified transaction $T^{\prime }$, which maintains the core meaning but has a syntactic alteration.

This modification is a strategic time such that it $T^{\prime }$ is propagated to certain non-adversarial nodes ($N^{\prime }$) before the legitimate transaction *T* can reach them. The race between $T^{\prime }$ them *T* creates a unique opportunity *A* to intervene in the signature verification process between *U* (the user) and $N^{\prime }$ (the honest nodes).

The altered transmission of the adversary can be sent to the target nodes and, therefore, creates a window through which the scam interactive signature can be counterfeited. The communication between *A* and $N^{\prime }$ occurs under a probabilistic model, in which the adversary can create a legitimate signature, bypassing common signature validation schemes and enabling malleability attacks. The adversary’s success is ensured within polynomial time due to the network’s synchronization and timing vulnerabilities. By influencing the propagation of $T^{\prime }$, the adversary effectively delays their confirmation, taking control of the signature generation process. This creates the risk that transaction acceptance is manipulated even when it *T* is still being validated. The OracleTrust scheme is designed to achieve the following security goals:

(G1) Oracle data integrity and authenticity: manipulated oracle-fed data must be rejected before ledger admission.

(G2) Transaction oracle binding: any accepted transaction should be cryptographically bound to the oracle commitment that created it.

(G3) Resistance to transaction malleability: an adversary cannot modify a transaction’s signature, metadata, or oracle payload without causing validation failure.

(G4) Robustness to oracle misbehavior: delayed reveals and inconsistent oracle reports should be penalized and excluded via the trust-scoring mechanism.

### Security analysis

In this section, we consider the security of the guarantees offered by OracleTrust according to the adversarial model introduced in the previous section. Our discussion is based on the assumptions about cryptographic tools and protocols that are presented in our design, which include commitment protocol (Eq. (11)), commit-and-reveal approach with integrity (Algorithm 2), transaction validation (Algorithm 3), signature validation and trusted set (Algorithm 1).

### Assumptions and target properties

Below are some of the assumptions made regarding these arguments:

The hashing function employed for determining the commitment in Eq. (11) is collision-free and preimage-resistant. The elliptic curve digital signature algorithm (ECDSA) that the oracles employ in signing their data is secure against the EUF-CMA attack. The blockchain consensus mechanism deployed is not susceptible to double spending and chain reconstruction attacks, and it is also said to possess finality attributes. Finally, the trust and penalties updating process occurs flawlessly, resulting in punishment by penalization of oracles that keep misbehaving until they are kicked out of the set of trusted oracles.

With these assumptions, OracleTrust aims to achieve the following security guarantees:

Security Guarantee 1 (oracle data binding): after an oracle commits to some value, it will not be able to later open the commitment value with a different data–secret pair that satisfies the integrity test.

Security Guarantee 2 (transaction non-malleability): an adversary cannot modify a valid bound transaction to a new one that satisfies the bound transaction verification process.

Security Guarantee 3 (source authentication): any transaction accepted by the network has to be authenticated by an oracle within the set of trusted oracles.

These three security guarantees align with the high-level objectives of oracle data integrity and authenticity (Objective G1), binding between transaction and oracle and protection against transaction malleability (Objective G2, Objective G3), and resilience against adversarial oracles (Objective G4).

### Oracle data binding and integrity

OracleTrust uses a hash-based commitment scheme in the commitment phase, as defined in Eq. (11). First, each oracle calculates a commitment using its data and local secret and then reveals the same pair in the reveal phase. In Algorithm 2, the commitment is recalculated using the revealed values, and a comparison is made between the calculated commitment and the commitment stored during the commitment phase. A successful comparison and satisfaction of the timing constraints lead to the acceptance of the reveal.

Let us assume that an adversary tries to break Property 1 by publishing a commitment value and revealing it to a different pair of data and secret that also satisfies the integrity constraint. For this, he needs to find two different inputs that will produce the same hash value as given in Equation (11). However, due to the properties of collision resistance and preimage resistance of the hash function, the chance of this occurrence is negligible. Thus, any commitment that passes through Algorithm 2 is tied to a unique pair of data and secret committed at the commitment phase.

### Transaction binding and non-malleability

A bounded transaction in OracleTrust is described as a tuple consisting of the transaction content, metadata, the commitment created from these elements, the signature on this commitment by the oracle, and the set of trusted oracles at that moment (the exact form of this tuple is given in the design section and used in Algorithm 3). There are two main steps involved in the verification process for such a bounded transaction:

Firstly, the validator derives the commitment using the provided transaction and metadata and makes sure that the generated commitment matches the committed one. This ensures no changes have been made to either of these elements after the original commitment was computed. Finally, the validator executes Algorithm 1 for checking the ECDSA signature of the commitment and obtaining the public address of its signer, which must be in the trusted oracles set.

An adversary wishing to malleate a transaction has essentially two options. First, it may try to modify the transaction body or its metadata after the transaction has committed. In that case, the recomputed commitment will differ from the committed value except with negligible probability. Passing the commitment equality test in Algorithm 3 despite changing the underlying data would require finding a second input that hashes to the same output as before, which is precisely a collision or second-preimage attack on the hash function. Under stated assumptions, this is infeasible.

Second, the adversary may decide to keep the commitment fixed but change the authorization. To do so, it would have to produce a new signature on the (now fixed) commitment that verifies under some oracle address, preferably in the trusted set, without access to that oracle’s private key. This is exactly the kind of attack ruled out by the EUF-CMA security of ECDSA: producing a fresh, valid signature on a message for a key controlled by someone else is infeasible.

The fact that a modification in any way of either the transaction itself or its metadata invalidates the commitment test, as well as the fact that an attempt to authorize a new commitment using anything other than the oracle’s secret key breaks signature unforgeability, makes it impossible for the adversary to come up with an alternative transaction that can pass Algorithm 3.

### Source authentication and trusted set semantics

Algorithm 1 verifies that the commitment was signed by a legitimate oracle. Given a commitment and a purported signature, the algorithm verifies the signature, recovers the signer’s address from the signature, and checks whether the address is in the trusted oracle set maintained by the system.

If the signature is valid, ECDSA’s properties ensure that the recovered address is the unique public key that generated the signature for the given commitment. The final membership test against the trusted set then enforces that only oracles that have not been demoted by the trust mechanism can authorize bound transactions. Any failure at either step results in the transaction being rejected. Thus, any accepted transaction is guaranteed to be authorized by an oracle in the trusted set, and any transaction not backed by a trusted oracle cannot be admitted. Combined with the unforgeability of signatures and the data-binding already established, this yields Property 3 (source authentication) and contributes to both transaction-oracle binding and to robustness against untrusted oracles (Goal 4).

### Robustness against misbehaving oracles

OracleTrust does not depend on the static inclusion or exclusion of the oracles. It also includes a trust scoring and penalty system based on the equations described in the design section. Each oracle is assigned a penalty value and a trust score. The value of the penalty and the trust score are updated based on the oracle’s behaviour in each round:

Late reveals and violations of the time constraint increase the penalty value.

Integrity violations, such as the failure of the integrity check in Algorithm 2, also impose a penalty on the oracle;

Correct and timely contributions increase the trust score.

As the system progresses, oracles with a history of misbehavior are assigned a higher penalty value, which decreases their trust score. As a result, the oracle is removed from the trusted set used by Algorithm 1 and Algorithm 3. On the other hand, honest oracles maintain or increase their trust scores.

Since transaction validation explicitly requires signatures from addresses in the trusted set, the impact of misbehaving oracles is reduced over time. The effectiveness of any attack based on strategic delay, strategic revelation, or sporadic misreporting is reduced over time. The reason is that the system adapts to the oracle’s misbehavior by reducing its authorization power. This is the basis for the system’s long-term security as described in G4.

### Attack vectors and overall security

Aside from transaction malleability and oracle misbehavior, there are a few other attack scenarios that are mitigated through the design of OracleTrust. The commitments are derived from the content of a transaction and related metadata. Reusing previous Oracle output in a new transaction context will, in general, yield a different value or violate timing constraints and thus fail the commitment or timing tests. Oracle collusion/sybil attacks. Even in the case of oracle collusion or a sybil attack, where a single adversary controls multiple oracle identities, all identities must pass the cryptographic tests as well as have a high enough trust score. The trust and penalty update rules ensure that such patterns of adversary behavior are reflected in decreasing trust scores, thereby limiting the combined influence of colluding or sybil oracles within the trusted set. OracleTrust signs and verifies a constant value for a commitment, rather than a malleable value for a transaction that can have multiple possible encodings. As a result, traditional malleability attacks on digital signatures, in which a forger attempts to produce a valid signature on a message that was not signed, are not applicable. Indeed, any valid signature on a message other than the original commitment value violates ECDSA unforgeability, as does a valid signature on the original message without access to the oracle’s private key. The probabilistic analysis of the underlying blockchain consensus process, as presented in the preliminaries, provides a bound on the probability of successful attacks that combine transaction tampering with double-spending or chain rewriting attacks. So long as the adversary possesses less than the majority of consensus power and operates below the security level, the likelihood of any transactions that have been compromised or are fraudulent to survive both the OracleTrust authentication mechanism and the consensus process will be minimal. Based on the provided assumptions and threat model, a hash commitment scheme, bound transaction verification, signed transactions through a trustworthy list of oracles, and adaptive scoring of trust provide excellent, formally justified assurances of oracle data authenticity, oracle-transaction binding, transaction malleability resistance, and protection from oracle-based attacks.

### Proposed scheme

In this section, the smart contract within the proposed model, which serves as the core validation engine for verifying both oracle data and the transactions submitted to the blockchain, consists of the following phases: commitment phase, reveal phase validation, time constraint enforcement, reveal success rate, and penalty model for invalid reveal. For clarity, the notation used in this framework is shown in Table 2.

**Table 2:** Notation definitions.

Symbol	Definitions
*D*_*i*_	Data submitted by Oracle *O*_*i*_ during the reveal phase
*s*_*i*_	Cryptographic salt generated by Oracle *O*_*i*_
*C*_*i*_	Commitment value: $C_{i}=h(D_{i}allels_{i})$
*T*_*c*_	Deadline time for commitment submission
*T*_*r*_	Deadline time for reveal submission
*I*_valid_	Returns 1 if $h(D_{i}allels_{i})=C_{i}$, else 0
$\Delta T_{\text{max}}$	Maximum allowed commit-reveal time difference
τ	Minimum valid reveal ratio threshold (τ∈(0,1))
*T*	Set of trusted oracle public keys
*T*_*x*_	Transaction data
σ	Random salt/nonce for transaction
*C*	On-chain stored commitment value
keccak( *x)*	Keccak-256 hash function
(*v*, *r*, *s*)	ECDSA signature parameters
ecrecover( *h, sig)*	Ethereum signature recovery function

**1) Commitment Phase:** Let the commitment *C*_*i*_ generation process be given as:


$$\begin{array}{c} C_{i}=h(D_{i}∥s_{i}) \end{array}$$ (11)

where *C*_*i*_ represents the cryptographic commitment created by the i-th oracle *O*_*i*_. The value of *D*_*i*_ indicates the data being submitted by the oracle, and *s*_*i*_ indicates a secret validated only to the oracle. *h* is a cryptographic hash function, and the operator ∥ signifies concatenation. This procedure ensures that the commitment *C*_*i*_ binds the oracle to both the data *D*_*i*_ and the secret *s*_*i*_ without revealing either to the other party. The commitment obtained with the hash function is unique, making it computationally infeasible for the oracle to alter the data *D*_*i*_ after the commitment is issued, and ensuring data integrity. The commitment also guarantees that the verifiability of the datum can be determined at some later point, when the value of *s*_*i*_ is revealed, allowing the submission of the oracle to be authenticated. Let $\delta_{i}$ denote a binary flag representing the timely commitment status of the *i*-th participant:


$$\begin{array}{c} \delta_{i}=\left\{ \begin{array}{cc} 1, & \text{if }T_{\text{commit}}^{\left(i\right)}\leq T_{c}\\ 0, & \text{otherwise} \end{array}\right. \end{array}$$ (12)

where $T_{\text{commit}}^{\left(i\right)}$ represents the actual commit time of the *i*-th oracle, and *T*_*c*_ is the commitment deadline.

During the commitment stage, each oracle *O*_*i*_ reports data *D*_*i*_, the information it provides to the blockchain. To guarantee the confidentiality and inviolability of this information, the oracle also creates a secret *s*_*i*_ that is held only by the oracle. This secret is combined with the reported data *D*_*i*_ and passed through a cryptographic hash function *h*(), yielding the commitment value *C*_*i*_. The commitment *C*_*i*_ is then securely submitted to the blockchain before a specified time *T*_*c*_, ensuring that it is stored safely and cannot be altered without providing the secret.

**2) Reveal phase validation:** During the reveal stage, every oracle *O*_*i*_ submits their data *D*_*i*_ along with the secret *s*_*i*_ to prove it is the originator of the commitment *C*_*i*_. The contract checks the correctness of the reveal by validating that the hash of the data and the secret match the previously submitted commitment. Let $I_{\text{valid}}(C_{i},D_{i},s_{i})$ denote the data integrity validator, as follows:


$$\begin{array}{c} I_{\text{valid}}(C_{i},D_{i},s_{i})=\left\{ \begin{array}{cc} 1, & \text{if }h(D_{i}∥s_{i})=C_{i}\\ 0, & \text{otherwise} \end{array}\right. \end{array}$$ (13)

where *h* is the cryptographic hash function, “is” *D*_*i*_ is the revealed data, “is” *s*_*i*_ is the secret, and *C*_*i*_ is the original commitment.

Let Ω show the trusted set of oracles, as follows:


$$\begin{array}{c} \Omega =\{O_{i}|I_{\text{valid}}(C_{i},D_{i},s_{i})=1\wedge \delta_{i}=1\} \end{array}$$ (14)

where $I_{\text{valid}}(C_{i},D_{i},s_{i})$ is the data integrity validator from equation (13), and $\delta_{i}$ is the timely commitment status of the *i*-th participant from equation (12).

Only oracles that are both valid and committed on time are included in the trusted set Ω.

**3) Time Constraint Enforcement:** Let the time difference between reveal time and commitment time be constrained as follows:


$$\begin{array}{c} 0<T_{r}-T_{c}\leq \Delta T_{\text{max}} \end{array}$$ (15)

where *T*_*r*_ represents the reveal time when oracles submit their data and secrets, *T*_*c*_ represents the commitment deadline time, and $\Delta T_{\text{max}}$ is the maximum allowed time window between commitment and reveal. A reveal is only accepted if it occurs within this specific time window. The time difference between the reveal time *T*_*r*_ and the commitment time *T*_*c*_ must satisfy the conditions in Equation (15).

Let $\pi_{i}$ represent the delay flag of late reveals, as follows:


$$\begin{array}{c} \pi_{i}=\left\{ \begin{array}{cc} 1, & \text{if }T_{r}^{\left(i\right)}-T_{c}>\Delta T_{\text{max}}\\ 0, & \text{otherwise} \end{array}\right. \end{array}$$ (16)

where $T_{r}^{\left(i\right)}$ denotes the reveal time of the *i*-th oracle, *T*_*c*_ shows the commitment deadline time, and $\Delta T_{\text{max}}$ is the maximum allowed time difference between reveal and commitment. The delay flag, denoted by $\pi_{i}$, is used during the trust scoring algorithm to impose penalties on oracles that display their data after the maximum permitted time, and this directly influences the trust scoring algorithm of the oracles in Algorithm 2, where those oracles with a reveal time exceeding the maximum threshold receive a lower trust score. This time constraint ensures that the oracles release their information on time and prevents manipulation through tardy disclosure or front-running assaults. This time constraint helps ensure that every oracle is involved on time and truthful, thereby guaranteeing the integrity of the data that is promised on the blockchain.

**4) Trust scoring with continuous values:**

Let $\rho_{i}$ percentage computation is delay percentage computation is formally expressed as:


$$\begin{array}{c} \rho_{i}=\max\left(0,\frac{T_{r}^{\left(i\right)}-T_{c}}{\Delta T_{\text{max}}}\right) \end{array}$$ (17)

where $T_{r}^{\left(i\right)}$ represents the reveal time at which the *i*-th oracle has released information and secret to the blockchain to be verified, while *T*_*c*_ is the commitment deadline time by which all original commitments must be made to the blockchain. The parameter maximum time window at which the commitment should be made and the data revelation is defined by the parameter of maximum allowable time window, which is denoted as $\Delta T_{\text{max}}$. The max(0,·) function is used to make sure that negative values (early reveals) are counted as perfect timeliness. It is a delay measure expressed as a percentage, a normalized measure of violation of the timeliness of an oracle in relation to the maximum duration, according to its delay.

The maximum allowable time difference, which is denoted by $\Delta T_{\text{max}}$, to defines the permissible delay between the commitment and reveal phases in OracleTrust and plays a critical role in strengthening temporal security guarantees. Although transaction malleability can be alleviated by cryptographic provenance binding and smart contract-enforced signature verification, the $\Delta T_{\text{max}}$ constraint complements these mechanisms by addressing time-based attack vectors. Specifically, it $\Delta T_{\text{max}}$ limits the window in which oracle data can be revealed, thereby preventing replay attacks and the reuse of stale or delayed oracle messages. Setting $\Delta T_{\text{max}}$ too conservatively may lead to the rejection of valid oracle submissions due to benign network latency, whereas excessively large values could weaken temporal consistency and facilitate replay-based manipulation. Accordingly, it $\Delta T_{\text{max}}$ is chosen to trade off protection against replay and delayed-reveal attacks and the practical latency properties of decentralized blockchain networks, such that both integral information of transactions as well as the oracle information are maintained. The individual trust score of each oracle is then calculated, based on a multi-level bucket system which assigns continuous trust values, but still has a binary acceptance decision, as in equation (18), as follows. [55,64,65].

Let the trust score Trust_*i*_ for each oracle be calculated using a single trust vector that contains all possible trust values so that the numerical values do not need to be hard-coded in a formula:


$$\begin{array}{c} {\text{Trust}}_{i}=I_{\text{valid}}(C_{i},D_{i},s_{i})\times 𝐓[\text{bucket}(\rho_{i})] \end{array}$$ (18)

where $I_{\text{valid}}(C_{i},D_{i},s_{i})$ serves as the data integrity validator, returning 1 if the hash of revealed data *D*_*i*_ with secret *s*_*i*_ matches the original commitment *C*_*i*_, and 0 otherwise. The multiplication with delay-based trust weights ensures that only valid data receives positive trust scores. The trust vector $𝐓=[T_{1},T_{2},T_{3},T_{4}]=[0.95,0.80,0.40,0.00]$ contains the strategically assigned trust values, where the $\text{bucket}(\rho_{i})$ function determines the index based on delay percentage: bucket = 1 if $\rho_{i}\leq 0.10$, bucket = 2 if $0.10<\rho_{i}\leq 0.25$, bucket = 3 if $0.25<\rho_{i}\leq 0.40$, and bucket = 4 if $\rho_{i}>0.40$. Thus, oracles with delay percentages up to 10% (minimal delay) receive a *T*_1_ = 0.95 score representing high-reliability performance; those delaying between 10%–25% obtain a *T*_2_ = 0.80 score indicating medium reliability performance; oracles with 25%–40% delay get a *T*_3_ = 0.40 score for low-reliability performance; and any oracle exceeding 40% delay receives a *T*_4_ = 0.00 score denoting unreliable performance. This graduated approach enables a nuanced trust assessment that differentiates between varying levels of timeliness.

Let γ denote the global trust score, defined as the average trust level of all participating oracles in the current validation batch. This metric provides a comprehensive assessment of overall system reliability by considering both data validity and timeliness aspects across all oracles. For each oracle *i* (where *i* ranges from 1 to *n*), the individual trust score Trust_*i*_ is first calculated based on its data validity and timeliness, as defined in Equation (18). The global trust score, which represents the collective reliability of all oracles in the current batch, is then computed as the arithmetic mean of these individual scores:


$$\begin{array}{c} \gamma =\frac{1}{n}\sum_{i=1}^{n}{\text{Trust}}_{i} \end{array}$$ (19)

where *n* represents the total number of oracles actively participating in the current validation round and Trust_*i*_ represents the individual trust score of the i-th oracle. The resulting γ value serves as a comprehensive measure of the overall reliability of the batch and the system in general, with a value of near 1.0 indicating a very reliable collection of oracles.

To maintain data quality standards, OracleTrust enforces a minimum trust threshold for data acceptance. Let’s *A*_*i*_ denote the binary acceptance decision for the *i*-th oracle, as follows:


$$\begin{array}{c} A_{i}=\left\{ \begin{array}{cc} 1, & \text{if }{\text{Trust}}_{i}\geq \tau \\ 0, & \text{otherwise} \end{array}\right. \end{array}$$ (20)

where Trust_*i*_ is the individual trust score of the oracle *i* from Equation (18), and τ is the minimum trust threshold for data acceptance. For behavioral enforcement, a proportional penalty system is employed. Let *P*_*i*_ represent the final penalty amount imposed on the *i*-th oracle, as follows:


$$\begin{array}{c} P_{i}=B\times (1-{\text{Trust}}_{i}) \end{array}$$ (21)

where *B* denotes the fixed base penalty amount predefined in the smart contract configuration, and Trust_*i*_ is the trust score from Equation (18). The parameter *B* is a configurable constant that defines the maximum penalty amount. The computed value *P*_*i*_ represents the final penalty imposed on the oracle, which scales linearly with the degree of trust violation. This proportional approach ensures fair punishment; minor infractions receive smaller penalties, while major violations incur substantial consequences.

Let OTS_*i*_ maintain long-term oracle reputation through the Oracle trust score:


$$\begin{array}{c} {\text{OTS}}_{i}=\sum_{j=1}^{k}\left({\text{Trust}}_{i}^{\left(j\right)}\times w_{1}-P_{i}^{\left(j\right)}\times w_{2}\right) \end{array}$$ (22)

where *k* defines the number of recent interactions considered in the sliding window for reputation calculation. The term ${\text{Trust}}_{i}^{\left(j\right)}$ signifies the trust score of the *i* -th oracle in the *j* -th historical interaction, and $P_{i}^{\left(j\right)}$ represents the corresponding penalty amount. The weighting factors *w*_1_ and *w*_2_ balance the influence of trust achievements against penalty infractions, with the constraint that $w_{1}+w_{2}=1$. The resulting ${\text{OTS}}_{i}$ provides a comprehensive long-term Oracle Trust Score that evolves with the oracle’s behavior across multiple interactions, enabling the system to identify consistently reliable participants and filter out persistently poor performers. The *w*_1_ and *w*_2_ in Eq. (22) are configurable weighting factors that balance an oracle’s recent trust performance against its penalty history. These weights satisfy the normalization constraint $w_{1}+w_{2}=1$. In this framework, *w*_1_ governs the contribution of positive trust accumulation from valid and timely revelations, while *w*_2_ controls the impact of penalties imposed for malicious or faulty behavior. These are the more important weights, which are not constants but system parameters. This design permits OracleTrust to follow various deployment scenarios—e.g., an application prioritizing absolute data integrity might set $w_{1}>w_{2}$, or an application that favors liveness may choose the balance appropriately.

### Cross chain adaptability

Although OracleTrust is tested using the Ethereum platform, its architecture of OracleTrust is designed to be blockchain agnostic. This aspect has been clearly presented in related work section of this paper. In other words, blockchain platforms such as Polkadot and Solana have used a unique consensus mechanism. In a similar manner, OracleTrust architecture will be affected by such characteristics. The trust computation engine of OracleTrust is independent of the unique consensus rules of any blockchain system. The components of Oracle commitment, validation, and reputation update are agnostic to the blockchain system. In order to deploy OracleTrust in other blockchain platforms, only the components of OracleTrust related to smart contract interface and cryptographic binding need to be modified. Trust evaluation and adversarial mitigation components of OracleTrust remain the same. In particular, in a multi-chain system that uses shared security or a parallel execution architecture, OracleTrust can be used to maintain trust aggregation policies. In addition, verification procedures can be mapped to a target platform’s execution model. In other words, OracleTrust can be mapped to a heterogeneous blockchain platform without compromising its robustness against oracle-based transaction manipulation.

### Smart contract implementation

OracleTrust employs smart contracts, which are Ethereum-compatible and use these as the on-chain enforcement mechanism to compute oracle validation and authorize transactions. These contracts are the gatekeepers of the system, and only valid oracle data will be added to the blockchain, and oracle performance is monitored over time. The smart contract functions are designed to:

1) **Commitment submission:** Oracles submit their data along with a cryptographic commitment (a hash derived from their data and a secret). This commitment is stored on the blockchain and cannot be altered, ensuring the integrity of the data.
2) **Reveal verification:** During the reveal phase, the oracle provides the revealed data along with the secret used for commitment. The smart contract compares the hash of the revealed data with the stored commitment hash. If they match, the data is considered valid.
3) **Timing constraints:** The contract enforces $\Delta T_{\text{max}}$, ensuring that oracles reveal their data within the allowed time window. If the reveal is late, penalties are applied, and the oracle’s trust score is updated accordingly.
4) **Trust score and penalty updates:** Based on the timeliness and data validity of the oracle’s performance, the smart contract updates the oracle’s trust score (as per Algorithm 2). Oracles that frequently fail to reveal data on time or provide invalid data accumulate penalties and may be removed from the trusted set.
5) **Transaction authorization:** The smart contract allows the transaction to be approved after the check of the commitment and the compliance with the timing requirements. The signature validation is done on-chain through the Ethereum ecrecover mechanism, which means that only oracles in the current set of trusted entities can sign and authorize transactions. This cryptographic method ties the transaction to the data itself, thereby preventing transaction malleability.

The OracleTrust smart contract design, by moving off-chain data preparation and preprocessing to the oracle and on-chain lightweight verification and enforcement, reduces gas waste while maintaining a high level of security. This design enables this system to be scalable and efficient with an increase in the oracles and transactions.

### Prototype implementation and testing

The OracleTrust smart contracts prototype was written in Hardhat development framework. The smart contracts were compiled and locally simulated using Hardhat. It also enabled functional testing of the commitment-reveal mechanism and penalty enforcement to ensure that the system behaved as expected under various conditions. Deployment followed standard Ethereum-compatible workflows, ensuring that the smart contracts could be easily deployed on any Ethereum-based network. The testing was done in a local environment, simulating Oracle behavior and network delays to validate the robustness of the system under different scenarios.

### Platform selection and justification

The choice of Ethereum as the main platform for the implementation and testing of OracleTrust is due to several important factors. Firstly, Ethereum is a very popular decentralized application ecosystem with a great number of important decentralized financial applications and oracle services, which makes Ethereum the best option for checking the effectiveness of OracleTrust.

Secondly, the smart contract technology offered by Ethereum network perfectly suits OracleTrust validation principles, providing a stable platform for OracleTrust testing. Thirdly, the practical use of Ethereum network in different decentralized financial applications makes it one of the most appropriate platforms for OracleTrust testing due to the importance of the sector in blockchain business. Fourthly, the high quality of transactions and the latest research carried out in the Ethereum network make it one of the most appropriate platforms for comparing OracleTrust with other oracle-based solutions.

**Fig 5:**
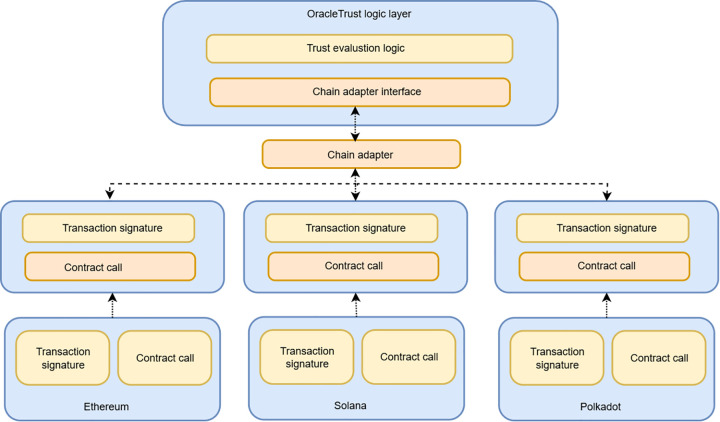
Cross-chain adapter architecture in OracleTrust.

As shown in Fig 5, OracleTrust keeps the chain-specific components separate from the main validation logic by using a cross-chain adapter. The logic layer talks to a single common interface. The adapter then turns these generic calls into the actual transaction signatures and contract calls for each chain, such as Ethereum, Solana, or Polkadot. If a new blockchain needs to be supported, only a new adapter module needs to be written; the trust evaluation logic of OracleTrust does not change. This abstraction layer provides OracleTrust with a clear path towards portability across heterogeneous blockchains such as Polkadot and Solana. In the present study, OracleTrust is implemented and evaluated on an Ethereum-compatible network. The main building blocks are provenance tracking, smart contract-based validation, and cryptographic commitment of oracle data, which use only standard tools such as hash functions, digital signatures, and programmable state updates. Because of this, the same validation logic can be moved to other platforms, while the adapter and on-chain part are adjusted to the native hash function, signature scheme and execution environment of each blockchain.

## Algorithm and analysis

In **Algorithm 1**, let *v* represent the recovery identifier, and (*r*,*s*) denote the ECDSA signature components. The algorithm also takes *m* as the input message and *T* as the trusted set of public addresses. In Ethereum, every signature consists of three parameters (*r*,*s*,*v*). Here, *v* plays a critical role in recovering the public key from the elliptic curve.

**Algorithm 1 Verify Signature with Trusted Set (m, sig, T)**

**Input:** Message *m*, signature *sig* = (*v*, *r*, *s*), trusted set *T*

**Output:** Boolean result of validation

1: (v,r,s)←sig /* Extract signature */

2: **if** *v* = 27 or *v* = 28 **then** /* Validate v is standard (27 or 28) */

3:    /* Valid v value, continue to signature verification */

4: **else**

5:    **return** 0 /* Reject non-Ethereum signature */

6: **end if**

7: h←keccak256(m) /* Compute message digest */

8: $h^{\prime }\leftarrow \text{eth\_prefix}(h)$ /* Apply Ethereum signing prefix */

9: $a\leftarrow \text{ecrecover}(h^{\prime },v,r,s)$ /* Recover signer address */

10: **if** *a* = null **then** /* Check if recovery was successful */

11:    **return** 0 /* Reject: invalid signature parameters */

12: **end if**

13: **if** a∈T **then** /* Verify signer is in trusted set */

14:    **return** 1 /* Accept: valid & trusted signature */

15: **else**

16:    **return** 0 /* Reject: signer is not trusted */

17: **end if**

As the elliptic curve points are symmetric, there exist two public keys whose value is identical to the same (*r*,*s*). The ambiguity is settled by the parameter that is given as *v*, which means which key among the two is to be used.

The validity of the recovery identifier is formally stated as follows: [64,66]:


$$\begin{array}{c} v\in \{27,28\} \end{array}$$

where the symbol “∈” represents set membership. This ensures that it *v* is strictly restricted to the two valid recovery identifier values permitted by Ethereum’s ECDSA signature scheme [67]. Any value *v* outside this range is deemed to be invalid and represents a non-Ethereum-compatible signature. In this case, the algorithm stops immediately and returns a rejection result (lines 2–6). These specific values, namely 27 and 28, are selected in the Ethereum legacy transaction format because they provide a clear distinction from other values in transaction encoding in order to enable deterministic extraction of public keys during signature validation. [64,66,68]. Assuming that the recovery identifier, denoted by *v*, is “valid,” the algorithm proceeds to compute the message digest of a message *m* defined by the Keccak-256 function:


$$\begin{array}{c} h\leftarrow \text{keccak256}(m) \end{array}$$

Ethereum adds a domain separation prefix so that signatures cannot be used as valid Ethereum transactions. The prefixed hash is computed as:


$$\begin{array}{c} h^{\prime }\leftarrow \text{eth\_prefix}(h) \end{array}$$

The Ecrecover function accepts four parameters: the prefixed message hash, $h^{\prime }$, the recovery identifier *v* (restricted to 27 or 28 in Ethereum’s legacy transaction format), and the ECDSA signature element, *r* and *s* [66,67,69,70]. In this case, it *r* is a representation of the *x* value of an elliptic curve point generated in the signature creation process, contributing to signature uniqueness, while *s* is a proof of ownership of a corresponding private key. The sender’s address is then obtained using the elliptic curve recovery function:


$$\begin{array}{c} a\leftarrow \text{ecrecover}(h^{\prime },v,r,s) \end{array}$$

If recovery is unsuccessful, i.e., where *a* equals null, the algorithm rejects the signature (lines 10–12). Otherwise, the address is validated against the trusted set *T*. This is achieved by the following indicator function, which equals 1 if the address is trusted, 0 otherwise:


$$\begin{array}{c} 1_{a\in T}=\left\{ \begin{array}{cc} 1 & \text{if }a\in T,\\ 0 & \text{otherwise.} \end{array}\right. \end{array}$$

Thus, the algorithm accepts the signature if it is both cryptographically valid and comes from a trusted address (lines 13–17).

In algorithm 2, the system processes a batch of oracles, validates their revealed data, ensuring their timely participation, and updates their reputation scores. The algorithm begins by initializing the counter for the total valid revelations, total_valid, and storing the total number of oracles, *n* (Lines 2–4). The algorithm then iterates over each oracle *O*_*i*_ (Line 5).

**Algorithm 2 Oracle Commitment-Reveal Validation with Trust Scoring**

**Input:** For each oracle *O*_*i*_: commitment *C*_*i*_, data *D*_*i*_, secret *s*_*i*_, commit time *T*_*c*_(*i*), reveal time *T*_*r*_(*i*), global parameters $\Delta T_{\text{max}}$, *B*, *w*_1_, *w*_2_

**Output:** Trust scores *Trust*_*i*_, penalty amounts *P*_*i*_, global trust score γ, updated oracle trust scores *OTS*_*i*_

1: sum_trust←0, n← number of oracles

2: **for** each oracle *O*_*i*_ **do**

3:    /* Phase 1: Validate Commitment-Reveal */

4:    **if** $h(D_{i}∥s_{i})=C_{i}$ **then**

5:       $I_{\text{valid}}\leftarrow 1$ /* Valid commitment */

6:    **else**

7:       $I_{\text{valid}}\leftarrow 0$ /* Invalid commitment */

8:    **end if**

9:    /* Phase 2: Calculate Delay Percentage */

10:    $\rho_{i}\leftarrow \max\left(0,\frac{T_{r}(i)-T_{c}(i)}{\Delta T_{\max}}\right)$ /* Eq. (17) */

11:    /* Phase 3: Calculate Individual Trust */

12:    $Trust_{i}\leftarrow I_{\text{valid}}\times \text{continuousTrustMapping}(\rho_{i})$ /* Eq. (18) */

13:    /* Phase 4: Assign Penalty */

14:    $P_{i}\leftarrow B\times (1-Trust_{i})$ /* Eq. (19) */

15:    /* Phase 5: Update Historical Trust Score */

16:    $OTS_{i}\leftarrow OTS_{i}+(Trust_{i}\times w_{1}-P_{i}\times w_{2})$ /* Eq. (20) */

17:    $sum\_trust\leftarrow sum\_trust+Trust_{i}$

18: **end for**

19: /* Phase 6: Calculate Global Trust Score */

20: $\gamma \leftarrow \frac{sum\_trust}{n}$ /* Eq. (21) */

21: **return** $\left\{Trust_{i},P_{i},\gamma ,OTS_{i}\right\}$ for all *i*

For every oracle, the first phase involves validating the commitment reveal, in which the hash of the revealed data and secret is compared to the original commitment *C*_*i*_. This comparison is performed in Lines 7–11 of the pseudocode. If they match, the validity flag *I*_valid_ is set to 1; otherwise, it is set to 0. The second phase of the validation checks the timeliness of the reveal, as shown in Lines 13–17 of the pseudocode. The time difference between the reveal and commitment for every oracle is compared to the maximum allowed window $\Delta T_{\max}$. If the reveal is delayed, the delay flag $\pi_{i}$ equals 1; otherwise, it equals 0. This ensures that the oracles do not receive an unfair advantage by delaying their reveal until certain external circumstances favour them.

The third phase computes an individual trust score $Trust_{i}$ for the oracle by multiplying the validity flag *I*_valid_ by the inverse of the delay flag $\left(1-\pi_{i}\right)$ (Line 19). This ensures that a trust score of 1 is given only if both the reveal was valid and on time. The algorithm also increments a counter total_valid by *I*_valid_ to be used later (Line 20). The fourth phase computes a penalty flag *P*_*i*_ (Lines 22–26), which is set to 1 if the reveal was either invalid or late, and 0 otherwise. Penalties are used to disincentivise untrustworthy oracles over time. The fifth phase updates the oracle’s long-term trust score *OTS*_*i*_ by adding the weighted difference between its current trust score and penalty (Line 28). In this phase, weights *w*_1_ and *w*_2_ can be used by the system designers to favor honest participation and heavily penalize malicious or unreliable behavior. This cumulative scoring method allows trust to accumulate over time by repeatedly doing things correctly and reducing it when things go wrong.

The sixth phase computes the global success rate for reveal operations, denoted by γ, which equals the total valid revelations divided by the total number of oracles (Line 31). This gives a broad perspective on the trustworthiness of all oracle operations in the round. The function then proceeds to return the set containing all trust scores, penalty flags, global success rate, and updated trust scores for all oracle operations (Line 32), which essentially constitutes the backbone of the provenance layer in incentivizing oracles to be honest and prompt in all rounds.

Algorithm 3 serves as the final validation gate for the transaction, ensuring it is cryptographically linked to the correct oracle information and issued from a trusted source. The algorithm takes the transaction data *T*_*x*_, the salt σ, the digital signature sig, the original commitment *C*, and the trusted set *T* as input. The algorithm output is a binary vector where 1 indicate a valid transaction and 0 represents an invalid transaction. The validation occurs in two primary phases.

**Algorithm 3 Validate bound transaction (*T*_*x*_, σ, *sig*, *C*, *T*)**

**Input:** Transaction data *T*_*x*_, salt σ, signature *sig* = (*v*, *r*, *s*), commitment *C*, trusted set *T*

**Output:** Boolean result (1 = valid, 0 = invalid)

1: /* Check that the transaction data matches the oracle’s commitment */

2: $h_{T}\leftarrow \text{keccak256}(T_{x})$ /* Hash the transaction data */

3: $C^{\prime }\leftarrow \text{keccak256}(h_{T}∥\sigma )$ /* Recompute the commitment using the salt */

4: **if** $C^{\prime }\neq C$ **then** /* Verify data integrity */

5:    **return** 0 /* Reject: Data has been tampered with */

6: **end if**

7: /* Verify the signature is from a trusted oracle for this commitment */

8: /* This is a call to Algorithm 1 */

9: is_valid_sig←Algorithm1(C,sig,T) /* Verify sig on commitment C */

10: **if** is_valid_sig=1 **then**

11:    **return** 1 /* Accept: Data is intact and signature is valid */

12: **else**

13:    **return** 0 /* Reject: Signature is invalid or not trusted */

14: **end if**

**Phase 1 (Lines 1–6):** In the first phase, the integrity of the transactional information is ensured. The algorithm begins by hashing the transactional information *T*_*x*_ using Keccak-256 to derive *h*_*T*_ (Line 2). It proceeds by recalculating the commitment value $C^{\prime }$ by hashing the concatenation of *h*_*T*_ and the salt value σ (Line 3). The derived value $C^{\prime }$ is matched with the original commitment value *C*, which is stored on the blockchain (Line 4). If the values do not match, the transaction is rejected immediately (Line 5), as it implies that the information has been tampered with by the time it reached the oracle for the original commitment value *C*.

**Phase 2 (Lines 7–14):** This phase involves the validation of the transaction’s authenticity and authorization. This phase is performed by invoking Algorithm 1 (Line 9), which is essentially a subroutine for signature validation. The key feature of this phase is that the transaction’s signature is validated against the original commitment *C*, rather than the transaction data *T*_*x*_. This essentially links the oracle’s signature to the actual data it originally committed to. The subroutine Algorithm 1 essentially validates whether the signature is a valid Ethereum signature for the commitment *C*, and whether the signer of the transaction, i.e., the oracle, is a member of the trusted set *T*. The outcome of this subroutine, i.e., is_valid_sig, is evaluated (Line 10). If it is 1, the algorithm returns 1, implying that the transaction is fully valid and trusted (Line 11). Otherwise, the transaction is rejected due to an invalid or untrusted signature (Line 13).

This two-step mechanism ensures that the transaction is only approved if the data within the transaction is sound, as well as authentic and authorized in terms of origin.

## Experimental setup

### Experimental environment and tools

The experimental setup used a Core i5-10400 processor at 2.90 GHz, an NVIDIA GeForce GTX 1650 graphics processing unit, and Windows 10 operating system. The experiment was carried out using the dataset from the Ethereum network, which was used in the previous studies [51,71–73]. The dataset contain information from Ethereum blocks, transactions, and smart contracts [74]. Specifically, the information in the block records includes the block number, timestamp, gas limit, miner, etc.; the information in the transaction records includes the transaction hash, sender/receiver, transaction amount, gas used, transaction fee, etc.; and the information in the smart contract records includes the contract address, bytecode, events, etc. This hardware and software setup allows for an effective environment to carry out an analysis of the transaction behavior of the Ethereum network and test the vulnerability of smart contracts to tampering attacks and the vulnerability of the designed evaluation smart contracts to transaction malleability attacks, utilizing a standard Ethereum development stack. Smart contracts were created and tested in Solidity (v0.8.20) and utilized the Hardhat framework (v2.22.1) for its strong local simulation, compilation, and testing capabilities [75]. Transaction malleability attacks are simulated by tampering with the s component of the ECDSA signature [51,72,73]. To approximate real-world decentralized environments, the simulation models heterogeneous oracle response delays, probabilistic adversarial behavior, and dynamically varying transaction loads. These factors are intended to emulate practical deployment conditions observed in distributed oracle networks.

Hardhat is chosen over tools such as Truffle or Remix because it has a better plugin ecosystem, faster compilation, and better integration with TypeScript and JavaScript. Additionally, it has powerful debugging tools. It enables the deployment and testing of contracts on a fully controlled blockchain simulation. This makes it easier to reproduce and diagnose any errors. The Hardhat Network, which is a blockchain node that runs in-memory, is used. This resets between tests and allows for advanced logging for transaction tracing. While it allows forking of Ethereum’s main network for realistic tests, it is used with simulated data for controlled experiments.

The experimental environment consists of Node.js version 18.x, Mocha + Chai for behavior-driven test automation, and the operating system environment that supports the installation of Windows 11, Ubuntu 22.04, or macOS. The test cases are executed using the JavaScript Harhat script. These simulations simulate 10,000 Ethereum transactions, out of which 50% of the transactions are tampered with by modifying the value of the s parameter in the ECDSA signature component through the bitwise XOR operation $s^{\prime }=s⊕1$. All the transactions are ECDSA-based SECP256k1, as specified by the Ethereum cryptographic protocol. The test cases consist of 100 transactions per batch, and the bound transaction validator smart contract authenticates them using salted SHA256 hashes, incorporating the tamper-detection logic in the Solidity code.

### Compared methods

To evaluate OracleTrust’s effectiveness in preventing transaction malleability, use these key existing schemes for comparison purposes. The first one is NIput [76], which protects the transaction identifier by hashing it first, then appending the signature script. The second one is the Interactive Incontestable Signature (IIS) [46], which replaces this with an interactive protocol between the owner of the transaction and a pre-specified block producer, for instance, and incontestable confirmation of the transaction. The final one is the Segregated Witness (SegWit) [47] scheme, one of the most widely used Bitcoin improvement protocols for preventing signature malleability, which segregated signature data from the transaction data. In the proposed approach, a provenance-based model is used to ensure transaction integrity by filtering out malleable transactions in the blockchain. The mechanism of commitment revealed through provenance tracking has been used in the proposed solution to methodically address malleability issues arising from information manipulation of information in transactions, thus providing a more specific approach to introducing transaction integrity.

Multi-signature scheme against malleability attacks in DeFi [15] is a multi-signature scheme designed to ensure security in decentralised finance applications against malleability attacks. This scheme requires several parties to sign and approve every transaction, ensuring that you disapprove of the altered transactions. However, even though this scheme is meant to enhance security in decentralized finance applications, it also brings in a level of complexity in terms of computation. This approach enhances security without the complexity of a multi-validation process. In this way, a high level of security is achieved without using multi-validation processes. Additionally, in the proposed system, the use of provenance-based validation to ensure that the provenance of transactional information is well documented and monitored mitigates the threat of maliciously altered information when there is more than just one validator.

Though the CoSi protocol [77] provides integrity and authenticity for authoritative statements in a decentralized system by witness cosigning, it does not address the particular problem of transaction malleability in blockchain systems or oracle trust. The proposed method, OracleTrust, improves upon this by providing a two-layer provenance-based signature verification method that enables transaction malleability via the oracle and enhances oracle trust via cryptographic commitment and validation. This provides an enhanced guarantees for the processing of transactions and integrity that is not tampered with during the execution of smart contracts. Despite their short signatures via Weil pairing [78], addressing malleability attacks on DeFi and the significance of multi-signature schemes as a countermeasure against incorrect selection of authority for witness cosigning in a decentralized system, the proposed provenance-based validation method is a better solution to malleability and oracle trust for Ethereum smart contracts and thus is in a highly advantageous position regarding the security of Ethereum transactions. Although Chainlink provides decentralized oracle feeds and data feeds, it fails to tackle the transaction malleability issue addressed by OracleTrust [79]. The experiments have demonstrated that OracleTrust has the potential to provide higher detection rates for malleable transactions and lower memory usage in comparison to Chainlink. Towards Trustworthy DeFi Oracles [80], the authors discuss various trust mechanisms in DeFi systems, focusing on reputation and staking-based approaches to ensure data accuracy. However, none of these approaches addresses the transaction-level of malleability, which is the key to avoiding oracle-driven interference in the blockchain environment. Comparatively, a new two-level system has been proposed by OracleTrust, which tracks the provenance to and binds transactions, thus preventing oracle-driven malleability before writing transactions onto the blockchain. The results indicate that OracleTrust provides stronger transaction integrity and security, and is a better solution for ensuring the security of Ethereum-based smart contracts than the oracles used in Towards Trustworthy DeFi Oracles.

Though the IoT-based DACC solution [81] focuses on providing access and securing data in an IoT environment with the assistance of trusted oracles, it does not address the oracle-driven malleability of transactions. However, OracleTrust addresses oracle-driven malleability and oracle data integrity by focusing on transaction-level binding, which is more relevant to securing blockchain transactions than the solution presented in [81]. The NIput scheme is based on traditional cryptography, providing security for transactions, and IIS is based on interactive proof protocols, providing non-interactive transaction validation in nature [46,82–84]. The main focus of the SegWit scheme is on providing resistance against signature malleability, but it is insufficient to resist oracle-driven malleability attacks [47]. The performance and effectiveness of the OracleTrust scheme are better than all the above systems, with a validity ratio (Vr) of 97.8% for contract transactions affected by MA.

Cryptographic systems that address unforgeability and non-repudiation are puncturable signatures and permissioned blockchain-based signature schemes. Permissioned blockchain-based signature schemes are a permissioned blockchain architecture that provides data integrity and security using bilinear mapping and pairing-based cryptography. This is a similar approach, though the OracleTrust is more efficient and quicker. In particular, OracleTrust has the lowest latency of 2.27 s, whereas the PBATAS would incur higher computational cost because it requires identity authentication protocols and access control measures to validate participants.

Puncturable Signatures is applied in the design of Proof-of-Stake (PoS) blockchain protocols and protects against long-range attacks. PS is quite strong in PoS, although OracleTrust provides the same non-malleability guarantee with optimized commitment-reveal validation to minimize signature processing time, which provides it with a competitive edge on transaction time. In contrast to PS, which is oriented towards the election and signing of a blockchain leader, OracleTrust directly leverages transaction-level malleability resistance at the level of a transaction, which provides a more universal approach to contract transactions.

The permissioned blockchain-based anonymous and traceable aggregate signature scheme for IoT illustrates how aggregate signature works in the aggregate signature scheme for IoT in terms of privacy and traceability features. Although it has good privacy features, OracleTrust has better performance in terms of scalability, with lower memory requirements than other similar IoT Oracle systems. Malleable SNARKs and Their Applications [34] discusses how to develop malleable SNARKs to enable efficient modification of SNARKs while maintaining indistinguishability from properly generated SNARKs. It is considered the best paper to introduce a new concept to improve blockchain-based schemes in terms of security and other features. It is excellent in terms of performance in improving transaction integrity and manipulation resistance with a high load, which is exactly what OracleTrust offers in contract transactions. Although Malleable SNARKs [34] focuses on the malleability of modification in proof schemes, OracleTrust offers malleability resistance in terms of transactions, which is best in terms of utilizing latency and integrity in a decentralized oracle environment.

The other relevant aspect of resilience to manipulation and integrity of the data in the decentralized systems is Keeping Authorities Honest or Bust with Decentralized Witness Cosigning [77] article, where the discussion is on decentralizing the cosigning to ensure that the transactions are validated by more than one source to prevent the situation where one can gain access to the information and make the necessary changes. Although witness cosigning is not OracleTrust’s primary task, it also provides the necessary data and resilience against malleability by ensuring that the Oracle data is bound to the blockchain transactions in a cryptographic manner. The level of the MA resistance of the OracleTrust can be viewed as comparable to the cosigning mechanisms that utilize the decentralized witness as described in the article, optimized for the latency and memory usage of the blockchain transactions. In comparison to the open data platform that is based on decentralized oracles, which focus on data validation and reliability,OracleTrust is a more direct solution in dealing with the issue of transaction malleability that is based on the oracle, given that there is a focus on the integration of a dual-layer framework that is based on provenance and the binding of signatures on transactions. This implies that prior to the recording of the data from the oracle in the blockchain, It can not be manipulated, and hence, a more specific solution is needed for the prevention of oracle data manipulation on the blockchain.

### Experimental parameter settings

In this experiment, different parameters were configured for the assessment of the performance, trust management, and tamper resistance of the proposed blockchain-based oracle system. Table 3 shows the parameters of the experiment. These parameters were configured for realistic scenarios, for the assessment of the robustness of the proposed system with respect to adversarial oracle behavior, and for the assessment of the ability of the proposed system for the detection of tampered transactions. These parameters also enable comparison of the proposed system with numerous test conditions.

**Table 3:** Parameter settings.

Parameter	Meaning	Value(s)
*P*	Source participation rate — percentage of active oracles for transaction validation	0.3, 0.5, 0.7, 1.0
*g*	Oracle interaction trust factor — impact of oracle trust on validation success	0.5
*M*	Transaction malleability rate — percentage of tampered transactions used to test resilience	30% log entries
*N*_*o*_	Total number of oracles in the decentralized oracle network	80
*f*	Tampering rate — proportion of invalid transactions for testing tampered data	20%
*B*	Batch transaction size — number of transactions processed per batch for validation	50–150 transactions per batch
*T* (bytes)	Transaction size — size of each blockchain transaction	200–400 B
*N*_*tx*_	Number of data-request transactions evaluated per experiment run	500–4000
*T*_*block*_	Block interval of the underlying blockchain	12 s
*d*_*net*_	Average network delay assumed in the simulation	150 ms
SHA-256	Cryptographic hash function used for commitment verification and for hasing	–
ECDSA	Digital signature algorithm used for secure transaction/oracle validation and for signature verification	–

In this regard, the participation rate of the source, denoted by *P*, is assigned values 0.3, 0.5, 0.7, and 1.0. This rate represents the percentage of the oracles that are active within the transaction validation batch. The participation rate is used to test different levels of oracle participation, and the effect of such participation on the successful validation of transactions and on the oracle is determined. The total number of oracles within the network is constant, denoted by *N*_*o*_ = 80, while the number of data request transactions varies between 500 and 4000, denoted by *N*_*tx*_. The Oracle Interaction Trust Factor, denoted by *g*, is set to 0.5, which controls the extent to which the oracle trust score affects the final validation decision. The transaction malleability rate, denoted as *M*, is set to 30% of log entries to generate malleable transactions and test how well malleability attacks are handled. Additionally, the blockchain is set to a block interval of *T*_block_ = 12 s and an average network delay of *d*_net_ = 150 ms. SHA-256 is chosen as a hash function for commitment *g*eneration and transaction integrity verification. ECDSA is chosen as a digital signature scheme for transaction verification and oracle verification.

The batch size *B* is set to 50–150 transactions per batch. The batch size was varied to evaluate system behavior under different workload condition. The transaction size, denoted *T*, is set to 200 and 400 bytes, which are typical sizes for a blockchain transaction. It is also done to test performance across different transaction sizes. The tampering rate, *f*, is set to 20% of transactions being invalid. The tempering rate was fixed at 20% to evaluate the system under a controlled proportion of invalid transactions. Latency, gas consumption, and validation success rate were measured across different oracle set sizes and transaction loads. To evaluate computational efficiency, gas consumption and validation latency were measured across different oracle counts and transaction rates. Further, adversarial stress tests for the system’s robustness are simulated by injecting up to 30% conflicting oracle responses per test. Furthermore, validation accuracy and computational cost were explored to understand the system’s performance under increased workload. Although the implementation was carried out in a controlled Ethereum-like environment, the range of parameters was chosen to represent a decentralized oracle service workload in a practical sense.

### Indicator definitions

To evaluate the performance of the proposed provenance and smart contract-based method, the following indicators are defined:

a) Malleable detection rate (*M*_*d*_):

This indicator measures the system’s effectiveness in detecting malleable transactions.


$$\begin{array}{c} M_{d}=\frac{N_{mc}}{N_{mc}+N_{mm}} \end{array}$$ (23)

where *N*_*mc*_ is the number of malleable transactions caught, and *N*_*mm*_ is the number of malleable transactions missed. A higher value of (*M*_*d*_) indicates better detection capability, with a value closer to 1 representing close to complete identification of malleable transactions.

b) Oracle verification (*O*_*v*_):

This indicator measures the accuracy of data verification from the Oracle submission.


$$\begin{array}{c} O_{v}=\frac{N_{verified}}{N_{verified}+N_{tampered}} \end{array}$$ (24)

where *N*_*verified*_ is the number of oracle-submitted values that passed commitment verification, and *N*_*tampered*_ is the number that failed. The higher value of (*O*_*v*_) indicates more reliable oracle validation and stronger resistance to tampered data.

c) Overall validity ratio (*V*_*r*_):

For testing the malleability of the transaction execution with respect to the malleability conditions, *validity ratio* *V*_*r*_ is used. In this case, *N*_*vs*_ denotes the number of valid, successful transactions unaffected by malleability, and *N*_*total*_ denotes the total number of transactions submitted in the system. The validity ratio is the proportion of successfully validated transactions relative to the total number of submitted transactions. An increased validity ratio indicates greater robustness, meaning the transactions are successfully validated and are not impacted by malleability.


$$\begin{array}{c} V_{r}=\frac{N_{vs}}{N_{total}} \end{array}$$ (25)

d) Transaction delay (*D*_*t*_):

This represents the time taken from transaction submission to validation.


$$\begin{array}{c} D_{t}=t_{validate}-t_{submit} \end{array}$$ (26)

where *t*_*submit*_ is the timestamp when the transaction was submitted, and *t*_*validate*_ is the time when the transaction was verified and recorded.

## Discussion of results

The experimental evaluation of OracleTrust is presented in this section, compared with NIput, IIS, SegWit, Bit2CV, and Chainlink-based validation. The evaluation focuses on resistance to transaction malleability (MA) attacks, validity ratio, cache usage, gas consumption, and signing and verification time for smart contracts. The results show that OracleTrust offers better resistance to transaction malleability attacks and a higher validity ratio than the schemes, with varying batch sizes and oracle participation rates. Additionally, the framework offers consumes less memory and gas in most Therefore, OracleTrust aTherefore, OracleTrust appears to offer a good trade-off between security and efficiency for Oracle validation in a decentralised manner. Addition of provenance verification and commit-reveal logic, additional aggregation overhead is incurred, woverhead is incurred, which may become more pronounced in deployment scenarios with large scale. Future work, OracleTrust’s efficiency, as well as its validation across more heterogeneous workloads.

### Resistance against MA on standard transactions

**Fig 6:**
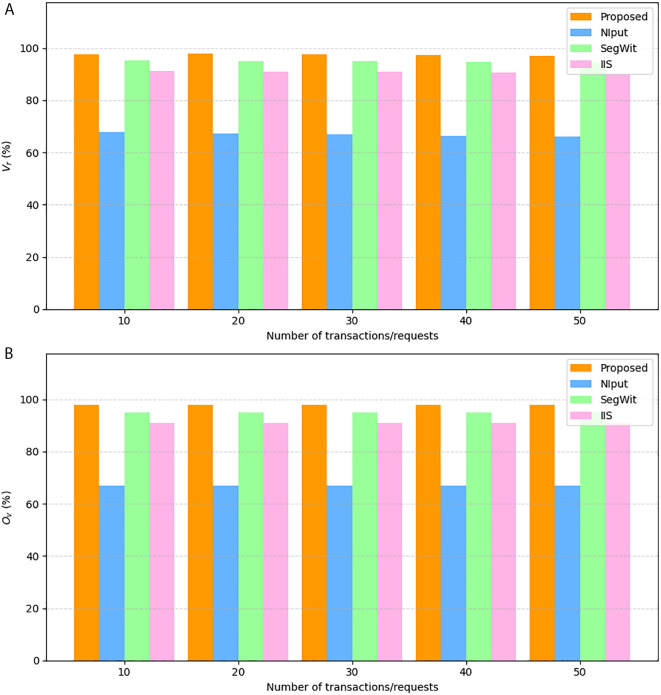
All Oracle verifications and transaction delay. **(A)**: Oracle verification comparison. **(B)**: Transaction delay performance.

Fig 6(A) illustrates oracle verification (*O*_*v*_) for the proposed approach for different batch sizes of transactions. The proposed scheme has achieved up to 97.2% honest transaction confirmations, with this figure varying swith this figure varying slightly across different batch sizes; 20 transactions and 97.1% for a batch size of 50 transactions. This figure clearly shows that the proposed scheme for provenance-bound verification sustains a near-complete level of MA resistance for regular transactions.

### Resistance against MA on contract transactions

Fig 6(B) indicates that the highest level of MA resistance is achieved with the proposed scheme across all batch sizes. However, the overall validity ratio (*V*_*r*_) is between 95% and 98% for all cases. For instance, when dealing with a batch size of |*T*| = 30 transactions affected by MA, the overall validity ratio (*V*_*r*_) is 97.8% using the proposed scheme compared with NIput, IIS, and SegWit.

### Latency for protocol execution

**Fig 7:**
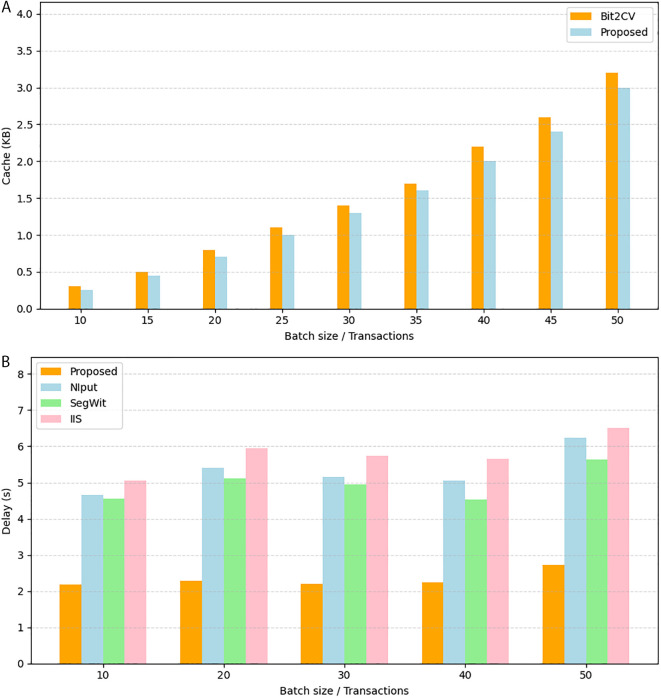
All validity ratios and cache space. **(A)**: Validity ratio comparison. **(B)**: Usage of cache space comparison.

The delay in transactions (Dt) is measured across varying batch sizes, as illustrated in Fig 7(A). The proposed scheme achieves the lowest transactions delay, averaging 2.27 achieves the lowest transaction delay, averaging 2.27 s across and IIS’s 5.88 s. Further, even when |*T*| = 50, the transaction delay in the proposed system is merely 2.72 s transaction delay in the proposed system is merely 2.72 s, less than half that of optimized commitment-reveal validation process that avoids unnecessary signature optimised while ensuring malleability resistance.

### Occupied space for contract transactions

Cache space requirements during protocol execution are shown in Fig 7(B). Comparing the proposed system with Bit2CV: At lower batch sizes, the cache space required is minimal for both systems (<0.5 *KB*). However, as batch size increases, the proposed method consistently requires less cache space than Bit2CV. At |*T*| = 50, the cache space required by the proposed design is 3.0 *KB*, while that required by Bit2CV is 3.2 *KB*. The proposed design achieves a saving of 6.25%.

Table 4 shows the comparison OracleTrust with existing schemes in terms of key reported performance metrics, including transaction delay, cache usage, signing time, and verification time. The results show that OracleTrust consistently outperforms the compared methods in several aspects. Specifically, it yields lower transaction delay than Niput, SegWit, and IIS, requires less cache usage than Bit2CV, and achieves better signing and verification efficiency than CoSi and BLS. Overall, the comparison highlights OracleTrust effectiveness in improving performance across diverse operational measures.

**Table 4:** Comparison of existing schemes with OracleTrust based on reported performance metrics.

Existing scheme	Metric	Existing	OracleTrust
NIput [45]	Transaction delay (s)	5.26	2.27
SegWit [83]	Transaction delay (s)	5.00	2.27
IIS [46]	Transaction delay (s)	5.88	2.27
Bit2CV [85]	Cache usage at |𝑇|==50 (KB)	3.20	3.00
CoSi [86]	Signing time (ms)	6–7	5–6
	Verification time (ms)	7–8	6.50–7
BLS [78]	Signing time (ms)	9–10	6–7
	Verification time (ms)	9–10	7–8

The performance of OracleTrust was tested under different participation rates of the oracle and different transaction volumes. Different load scenarios were considered to test the scalability of OracleTrust for different batch sizes and oracle interaction rates. The results show that OracleTrust can handle a large number of transactions with low latency and low memory consumption, thus proving its scalability for decentralized oracle networks.

**Fig 8:**
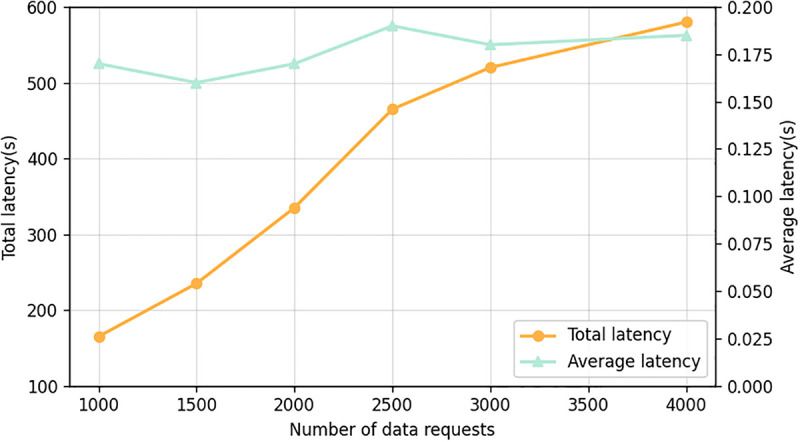
Total and average latency under increasing oracle workload intensity.

Fig 8 Total on-chain confirmation latency of OracleTrust as the number of Ethereum data request transactions increases from 500 to 4000. In this figure, the latency refers to the period between when a data request transaction is sent and when it is confirmed on the blockchain. While a total of 6000 off-chain data requests are used to simulate a high load on oracle services, only up to 4000 on-chain data requests are presented, as it has been observed that after 4000 on-chain transactions, the local Ethereum network gets saturated and the latency stabilizes.

Table 5 summarizes the workload-dependent latency characteristics of OracleTrust. The results indicate that the total execution latency increases monotonically with the number of on-chain data requests, rising from 165 s at 1000 requests to 580 s at 4000 requests. However, the average latency per request remains almost constant, fluctuating only between 0.17 s and 0.19 s. This behaviour suggests that the proposed framework exhibits stable request-level processing efficiency and controlled latency scaling as workload intensity increases. Therefore, OracleTrust demonstrates satisfactory scalability for higher transaction volumes without significant degradation in per-request response performance.

**Table 5:** Latency behaviour of OracleTrust under increasing workload.

Number of data requests	Total latency (s)	Average latency (s)
1000	165	0.18
1500	235	0.17
2000	335	0.18
2500	465	0.19
3000	520	0.18
4000	580	0.18

**Fig 9:**
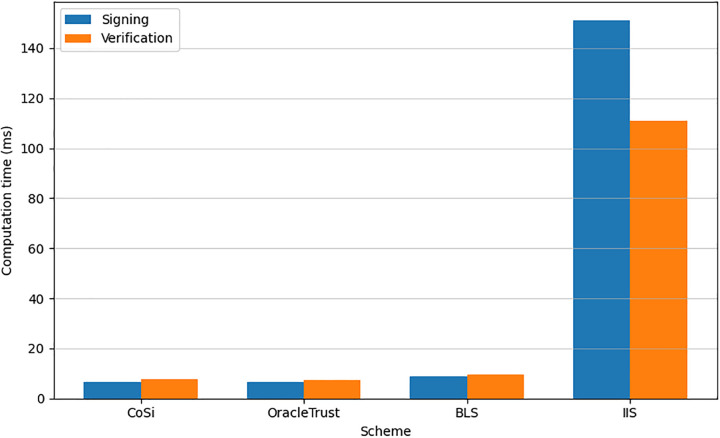
Computation time of signing and verification for CoSi, OracleTrust, and BLS schemes.

Fig 9 shows the computation times for the signing and verification operations in CoSi, OracleTrust, and BLS. OracleTrust’s computation are comparable to cosi, with the signing operation taking 6–7 ms and the verification operation taking 7-ms, both of which are lower than BLS (9–10 ms). This demonstrate that OracleTrust additional trust-aware logic incurs minimal overhead compared to Cosi and is more efficient than the BLS.

**Fig 10:**
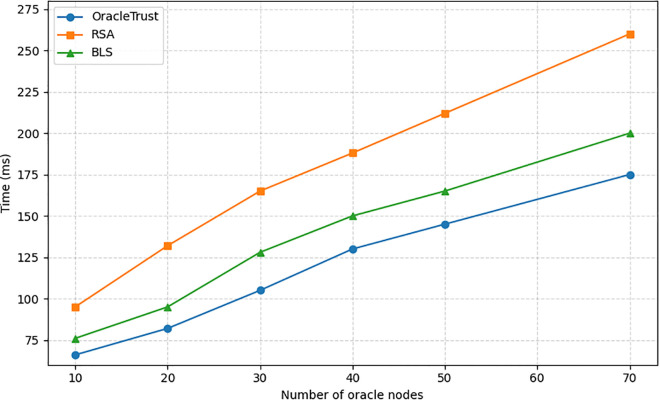
Required time of signing and verification for CoSi, OracleTrust, and BLS schemes.

As can be shown in Fig 10, the effect of the threshold configuration is shown as the number of participating nodes varies from 10 to 70. As the threshold value increases, a larger number of nodes is required to jointly contribute to a specific aggregated result. In other words, more trusted nodes must participate before a decision is made. This can be seen as an increase in security and level of consensus within a system, where a single malicious node or a small number of malicious nodes is not enough to affect a specific decision. However, a larger number of nodes is required to participate in a system, resulting in a moderate increase in overall processing time.

**Fig 11:**
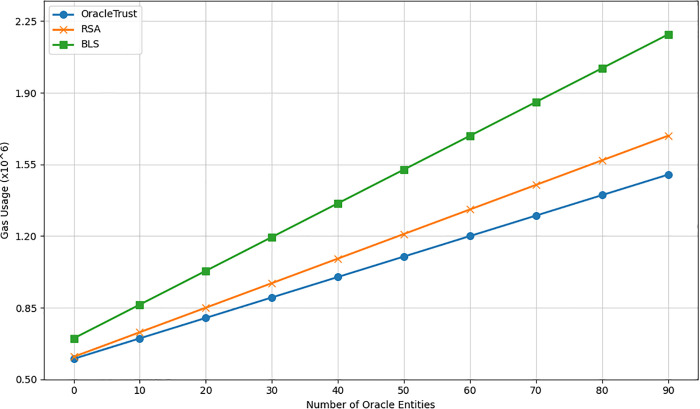
Comparison of GAS consumption for schemes.

The graph shown in Fig 11 compares the overall gas consumption for three oracle mechanisms: OracleTrust, RSA, and BLS, in relation to the overall number of oracle entities. It is evident that when the number of oracle entities is increased, a corresponding increase in overall gas consumption is seen for all three mechanisms.

OracleTrust shows the lowest gas consumption among all the test cases, indicating a better efficiency compared to the other oracle systems. The reduction in the cost of gas is critical for the deployment of the smart contract-based decentralized environment. However, in the case of RSA and BLS systems, a higher increase in gas consumption is noted when the number of oracle entities is increased. In both systems, BLS shows a higher level of gas consumption compared to RSA. The above observation shows how important it is to use an efficient oracle system like OracleTrust in a scenario where reducing gas costs is a critical requirement. In addition, it shows how trade-offs are made between security, scalability, and costs when an Oracle system is selected for a decentralized environment.

OracleTrust utilizes the threshold mechanism to balance security and node-level malleability and computational performance. As illustrated in Fig 11, even when the number of nodes (and hence the threshold value) is increased to a level of 70 nodes, OracleTrust is still able to attain lower aggregation times than RSA [87] and BLS threshold aggregation [87,88] while still satisfying the constraint that multiple trusted nodes are involved in the decision process. Ethereum in this study serves a more as a validation tool than a limiting factor. This is because of the inherent modular separation of trust computation and on-chain enforcement, which enables OracleTrust to be easily extended to support heterogeneous blockchains. This is because of its independence from the underlying consensus mechanisms, ensuring oracle evaluation, commitment verification, and adversarial mitigation strategies are consistent across different blockchain infrastructures. The experimental results demonstrate that OracleTrust offers low confirmation latency and high throughput across a wide range of workloads. As the number of data request transactions in each run increases from 500 to 4000, with batch sizes between 50 and 150 transactions, the latency curve increases gradually within a few seconds. This verifies that OracleTrust performs well in maintaining responsiveness after moving validation to the off-chain layer and only committing succinct proofs to the on-chain layer.

The parameter study on the participation rate *P* also verifies that OracleTrust works well even if only a portion of *N*_*o*_ = 80 oracles are active at any given time, which is in line with the dynamic oracle pool in decentralized oracle networks due to the dynamic nature of decentralized oracle networks in which nodes may be online or offline at different times. The experimental evaluation of OracleTrust in terms of validity preservation, protocol latency, cache efficiency, and cryptographic computation cost. The proposed framework attains a validity ratio of 97.8% for contract transactions under transaction-tampering conditions, which confirms the effectiveness of the provenance-bound verification mechanism in preserving transaction integrity. Furthermore, OracleTrust records an average transaction delay of 2.27 s and maintains a delay of 2.72 s at |𝑇|==50, indicating that the framework remains operationally stable as transaction volume increases. The observed cache usage of 3.0 KB at |𝑇|==50 further demonstrates that the design achieves strong protection with limited storage overhead. In computational terms, the signing time of 6–7 ms and verification time of 7–8 ms show that the added trust and provenance validation layers remain lightweight. These findings verify that OracleTrust achieves a favorable balance between security robustness, execution efficiency, and storage economy in decentralized oracle-assisted blockchain environments. From a security point of view, the results under the specified transaction tampering rate *M*, and the rate of oracle tampering *f*, indicate that OracleTrust can reliably identify and filter out a significant fraction of erroneous and manipulated transactions. The binding of oracle reports to on-chain transactions in a provenance-aware way helps to mitigate oracle-driven transaction tampering, and the trust factor *g*, along with the reputation update rules, helps to mitigate the effect of misbehaving oracles that have been consistently incorrect in their reporting. The proposed approach offers a viable way to incorporate oracle trust and transaction integrity in a unified validation process, which *g*oes beyond traditional methods for addressing transaction tampering and its limitations in explicitly addressing oracle behavior. From a security point of view, the results under the specified transaction malleability rate *M*, and the rate of oracle tampering *f*, indicate that OracleTrust can reliably identify and filter out a significant fraction of erroneous and manipulated transactions. The binding of oracle reports to on-chain transactions in a provenance-aware way helps to mitigate oracle-driven transaction malleability, and the trust factor *g*, along with the reputation update rules, helps to mitigate the effect of misbehaving oracles that have been consistently incorrect in their reporting. The proposed approach offers a viable way to incorporate oracle trust and transaction integrity in a unified validation process, which transcends traditional methods of addressin*g* transaction malleability and its limitations in explicitly addressing oracle behavior.

This evaluation focuses on an Ethereum-compatible environment, managing oracle workload and adversarial behavior. This enables the isolation of the impact of the design decisions, such as batching, participation rate, and the trust weighting. Though the experimental evaluation is performed on an Ethereum-compatible network, the on-chain validation of the OracleTrust workload is comparable to the typical smart contract operations that have already been benchmarked on heterogeneous public blockchains. Bistarelli et al. [22] evaluate equivalent smart contracts on Ethereum, Tezos, Polkadot, and Solana, reporting the gas cost of simple arithmetic, cast operations, and a healthcare-style record insertion operation. Their results indicate that, notwithstanding absolute gas requirements varying across these platforms, a common contract logic can be applied across all of these systems, and the costs will vary depending on the underlying runtime and fee model. Moreover, since OracleTrust’s logic for provenance and signature validation only depends on standard hash functions, signature verification, and state updates, and these are supported across these systems, this analysis indicates that this logic can, in principle, be applied across other programmable blockchain systems with similar relative costs. Finally, although native deployment of OracleTrust on these substrates and similar platforms is technically feasible, a full empirical re-evaluation of this logic on these systems is left as future work to broaden the scope of this work without changing the underlying design of OracleTrust.

### Conclusion and future work

In this research work, an OracleTrust smart contract scheme based on signature validation is presented, which prevents transaction malleability attacks through a provenance-driven approach. The proposed dual-branch, provenance-based smart contract, combined with cryptographic commitments, enhances security while reducing latency and efficient resource utilization compared to existing state-of-the-art approaches. Future work will focus on enhancing the OracleTrust trust management system by integrating federated learning to improve the detection of oracle misbehaviors such as commitment delays, reveal delays, and validation outcomes. With federated learning, the Oracle cluster will be trained to calculate trust scoring and slashing without altering the core commit-reveal process and transaction validation logic. This will strengthen OracleTrust as a data-driven approach, further improving its effectiveness. Furthermore, future research will includes deployment and testing OracleTrust on blockchain testnets and empirical validation across diverse blockchain ecosystems to further demonstrate its practical applicability.
